# Poultry handling-related stress alters membrane composition, extracellular vesicle production, survival, and virulence in *Campylobacter jejuni* Bf

**DOI:** 10.1128/aem.01265-26

**Published:** 2026-08-14

**Authors:** Jeanne Malet-Villemagne, Rochelle D'Mello, Yingxi Li, Zoran Minic, Karine Gloux, Florence Dubois-Brissonnet, Bastien Prost, Audrey Solgadi, Christine Péchoux, Vlad Costache, Marianne De Paepe, Zhu Zhang, Gilles Tessier, Jasmina Vidic

**Affiliations:** 1 Université Paris-Saclay, INRAE, AgroParisTech, Micalis Institute, Jouy en Josas, France; 2 Department of Chemistry and Biomolecular Sciences, John L. Holmes Mass Spectrometry Facility, University of Ottawa 6363, Ottawa, Ontario, Canada; 3 UMS-IPSIT SAMM Facility, Université Paris-Saclay, Inserm, Ingénierie et Plateformes au Service de l'Innovation Thérapeutique, Paris-Saclay, France; 4 MIMA2 Imaging Core Facility, Micalis Institute, INRAE, Jouy-en-Josas, France; 5 Université Paris-Saclay, INRAE, GABI 27048, Jouy en Josas, France; 6 Institut de la Vision, Sorbonne Université, CNRS-UMR 7210, Inserm-UMR S968 27063, Paris, France; 7 Université Paris Cité 555089, Paris, France; Universita degli Studi di Napoli Federico II, Portici, Italy

**Keywords:** *Campylobacter jejuni*, bacterial adaptation, oxidative and thermal stress, extracellular vesicles

## Abstract

We investigated the adaptation of the aerotolerant *Campylobacter jejuni* Bf strain to conditions mimicking the poultry slaughter process. We showed that *C. jejuni* Bf survives and actively multiplies under combined thermal and oxidative stress. Stress exposure induces cell rounding, loss of motility, remodeling of membrane composition, decreased membrane fluidity, and metabolic reprogramming with increased intracellular ATP levels. While maintaining the lipid composition of its plasma membrane, *C. jejuni* modulates the lipid composition of its extracellular vesicles when exposed to stress. Notably, stressed *C. jejuni* cells release extracellular vesicles with increased toxicity toward the epithelial barrier of Caco-2 cells, potentially facilitating invasion of the gut epithelium.

**IMPORTANCE:**

*Campylobacter* infections are one of the leading causes of foodborne gastroenteritis worldwide. *Campylobacter* readily enters the food chain and is transmitted to humans, primarily through the consumption of contaminated poultry meat. The high prevalence of aerotolerant human *Campylobacter jejuni* isolates suggests a correlation between their ability to survive under aerobic conditions, virulence, and resistance to harsh stress conditions. However, the underlying mechanism remains unclear. Our results show that *C. jejuni* extracellular vesicles are part of a survival strategy that links environmental adaptation with pathogenicity.

## INTRODUCTION

Bacterial infection remains a major challenge in medicine and public health. They are the second leading cause of death globally, after ischemic heart disease (1). Microbes establish infections and survive within their hosts through continual evolutionary adaptation. Additionally, adaptation across diverse biotopes, including animals, food, and water, can enhance bacterial infectivity and enable host switching (2, 3). Understanding microbial adaptation to consecutive stress within ecosystems, which allows pathogens to colonize and persist in their hosts, has profound implications for improving public health, as highlighted by WHO’s One Health approach.

*Campylobacter* infections are among the leading causes of foodborne gastroenteritis worldwide. As a commensal organism in the intestinal tracts of food-producing animals, *Campylobacter* readily enters the food chain and is transmitted to humans, primarily through the consumption of contaminated food products. Epidemiological studies estimate that 50%–80% of human campylobacteriosis cases originate from the poultry reservoirs (4, 5), with *Campylobacter jejuni* (~80%) being the leading strain causing human infections (*6*). Notably, the persistence of *C. jejuni* along the food chain presents a paradox, given its fastidious growth requirements under laboratory conditions and widespread occurrence in seemingly unfavorable environmental settings (7, 8).

Once chickens become colonized, *C. jejuni* rapidly spreads throughout the flock and persists in the intestinal tract until slaughter (9). During processing, leakage of intestinal material can lead to carcass contamination, with bacterial loads reaching up to 6.5 log CFU/g (5). In Europe, under the *Campylobacter* process hygiene criterion, monitoring of poultry samples found that 16.0% exceeded the limit of 3 log CFU/g (4). During poultry processing, *C. jejuni* is exposed to various stressors, including heat, cold shock, acid stress, and oxidative stress. Under acute exposure, spiral-shaped *C. jejuni* cells become coccoid owing to oxidative damage (10). This morphological change is one of the events that occur as bacteria enter a viable but non-culturable (VBNC) state, which hinders detection on conventional culture media during quality control procedures (11, 12). This phenomenon may explain the continued incidence of campylobacteriosis in developed countries despite advanced sanitation and food safety standards. At refrigerator temperatures, the membrane fatty acid composition of coccoid cells is almost identical to that of spiral cells, while intracellular ATP levels remain high (13). Visualization of VBNC *C. jejuni* cells in aging cell suspensions revealed that the outer membrane was lost (14). In addition, membrane instability caused by abiotic stress promotes vesicle budding to relieve cell envelope tension or to export damaged components (15). Bacterial production of extracellular vesicles (bEVs) is part of the adaptive and survival strategies in hostile environments, including during food processing or within the host (15–17).

To cope with abiotic stress, *C. jejuni* cells generate proteins in response to new conditions. Specifically, the bacterium produces heat shock proteins (e.g., GroEL, ClpB, and DnaK) in response to thermal and acid stress (18, 19), and detoxification enzymes (e.g., KatA, SodB, AhpC, Gpx, and Ccp) to counter oxidative stress (18, 20–22). The multifunctional protein polynucleotide phosphorylase (PNPase) enables the long-term survival of *C. jejuni* at common refrigeration temperatures (23). While earlier studies often examined only single types of stress (acidic, oxidative, or thermal), recent research has begun to investigate the impact of successive stresses encountered during poultry slaughter and processing (24). This approach can better predict *Campylobacter* behavior, as cell history plays an important role in adaptation by introducing physiological responses involved in co-selection mechanisms, including co-resistance, co-regulation, biofilm formation, or entry into a VBNC state (2, 25). While many stress conditions decrease ATP levels due to growth inhibition or metabolic slowdown, transient or mild stress conditions can induce short-term increases or stabilization of ATP levels associated with adaptive metabolic responses and subsequent behavior regarding additional stresses (19, 26, 27).

Resistance to environmental stress during processing varies among strains (28). Not all subtypes survive in the abattoir environment, and the genetic diversity of *Campylobacter* decreases through carcass chilling during processing (29). Importantly, cold-tolerant *C. jejuni* isolates usually exhibit elevated aerotolerance (30). The majority of *C. jejuni* clinical isolates and those from retail chicken meat show high aerotolerance and can survive more than 24 h of aerobic exposure (31, 32). Therefore, to decrease the prevalence of human Campylobacter infections, it is necessary to understand the mechanism of adaptation to environmental stress in aerotolerant *Campylobacter* strains (33).

Here, we show how consecutive stresses mimicking poultry processing steps affect the fitness and virulence of *C. jejuni*. We used the clinical Bf strain, which was previously shown to tolerate aerobic environments (34) and to survive starvation in sterile water by entering a non-coccoid VBNC state (35). We focused on structural, lipidomic, and proteomic cell adaptation to combined thermal and oxygen stress as well as the interaction of adapted cells with the human intestinal cell line Caco-2.

## RESULTS

### Viability of *C. jejuni* Bf under combined thermal and oxygen stresses

**Fig 1:**
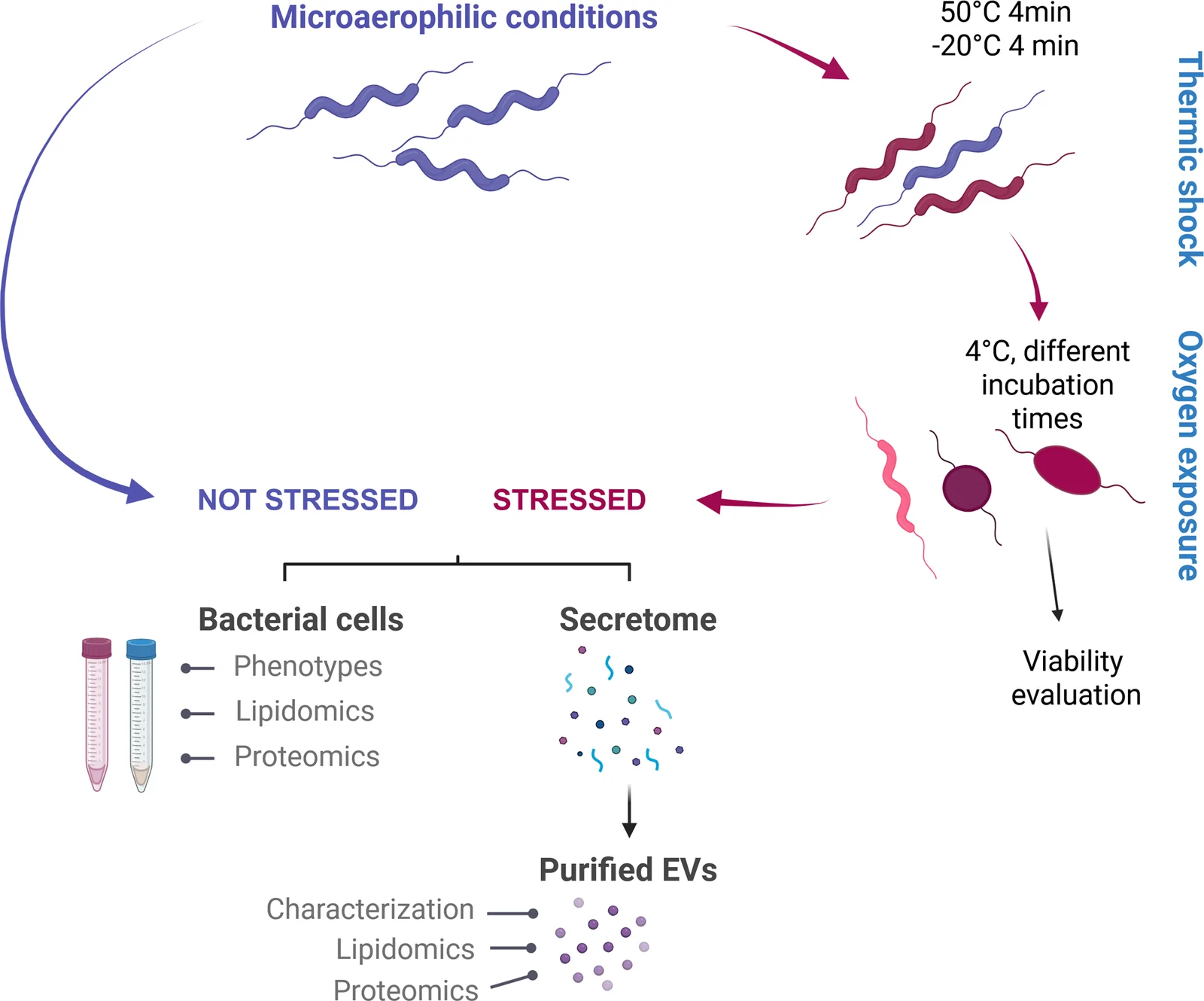
Schematic illustration of the tests performed in this study. The protocol established to mimic consecutive stresses encountered by *C. jejuni* during poultry slaughter included thermal and oxidative stresses. Stressed and unstressed bacterial cells, as well as their secretomes and bEVs, were characterized and analyzed for their lipidomic and proteomic contents.

*C. jejuni* encounters several stressors during poultry slaughter and processing, particularly during the scalding and chilling stages, which subject the bacterium to heat and cold stress, respectively. Subsequently, chicken cuts are conditioned and stored under a modified atmosphere, which can impose substantial oxidative stress. Therefore, we investigated the impact of sequential exposure to a hot water bath (50°C), freezing (−20°C), and oxygen exposure at 4°C, conditions that mimic poultry processing-related stresses, on the viability and morphology of the aerotolerant *C. jejuni* Bf (34, 35). Figure 1 illustrates the stress protocol used and the analyses performed in this study. Assuming that *Campylobacter* cells attached to chicken carcasses are not subjected to starvation conditions, and to avoid introducing additional stress associated with nutrient limitation, all tests were conducted in BHI broth.

**Fig 2:**
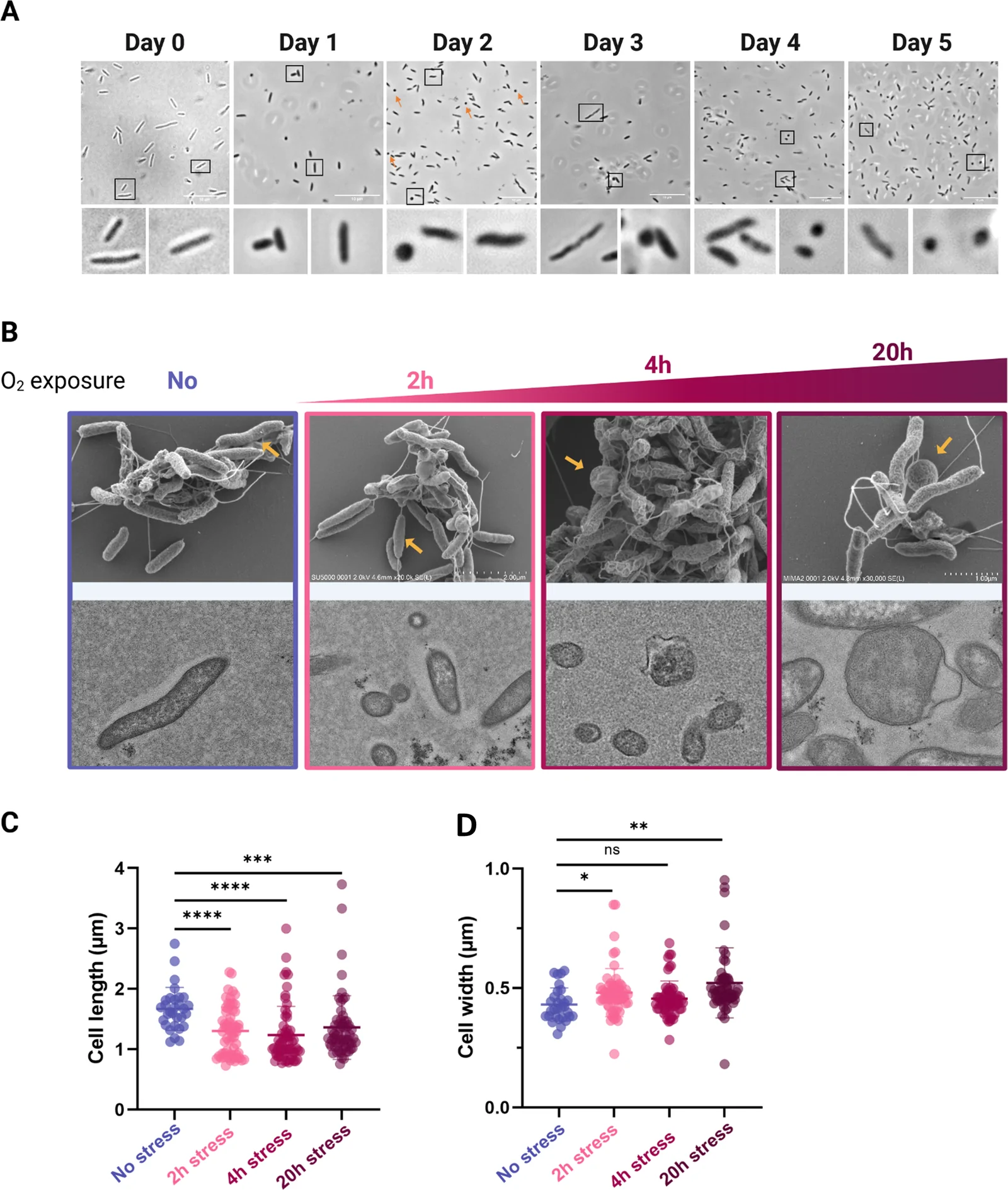
*C. jejuni* Bf survives combined thermal and oxidative stresses by changing its shape from spiral to coccoid. (**A**) Phase-contrast micrographs of *C. jejuni* in BHI over 8 days. Bacteria highlighted in black squares are shown enlarged in the bottom panels. (**B**) SEM and TEM images of *C. jejuni* Bf during the 20 hours following stress induction. (**C and D**) Cell length and roundness of *C. jejuni* Bf in BHI. Data are presented as mean ± standard deviation from independent experiments (*n* = 3). Statistical significance was determined by Student’s *t*-test; ns, not significant, *P < 0.05, ** *P* < 0.01, ***P < 0.001, ****P < 0.0001.

Phase-contrast images of *C. jejuni* were recorded over 1 week to track morphological changes in cells subjected to consecutive stress. The initial bacterial population (Day 0, Fig. 2A) consisted of typical curved or spiral-shaped cells that moved rapidly in helicoidal or corkscrew patterns through the liquid (Video S1). From the first day of incubation under aerobic conditions, coccoid forms appeared and increased in abundance over time at the expense of a spiral-shaped subpopulation (Fig. 2A). In addition, bacterial motility progressively decreased, and the cells became immobile, which facilitated microscopic observation (Videos S1 and S2). To confirm the morphological changes, we recorded scanning electron microscopy (SEM) and transmission electron microscopy (TEM) images of stressed *C. jejuni*. Untreated Bf cells displayed a single spiral turn form, as previously observed (35). After experiencing stress, coccoid-like cells were observed rapidly (Fig. 2B). This suggests that morphological adaptations in a subset of the bacterial population occur as early-stage protective mechanisms against stress. Interestingly, vacuoles were occasionally detected in coccoid cells (TEM cross-sections in Fig. 2B). Their presence may reflect either stress-related cellular alterations or a physiological adaptation mechanism. Previously, vacuolated coccoid cells were found in the VBNC state of *C. jejuni*, probably assisting the bacterium in compartmentalizing damage and shifting to a dormant morphology (36). Quantitative analysis of the population morphology confirmed this progressive shape transformation, as cell length decreased (Fig. 2C), whereas width increased (Fig. 2D) over time after treatment.

**Fig 3:**
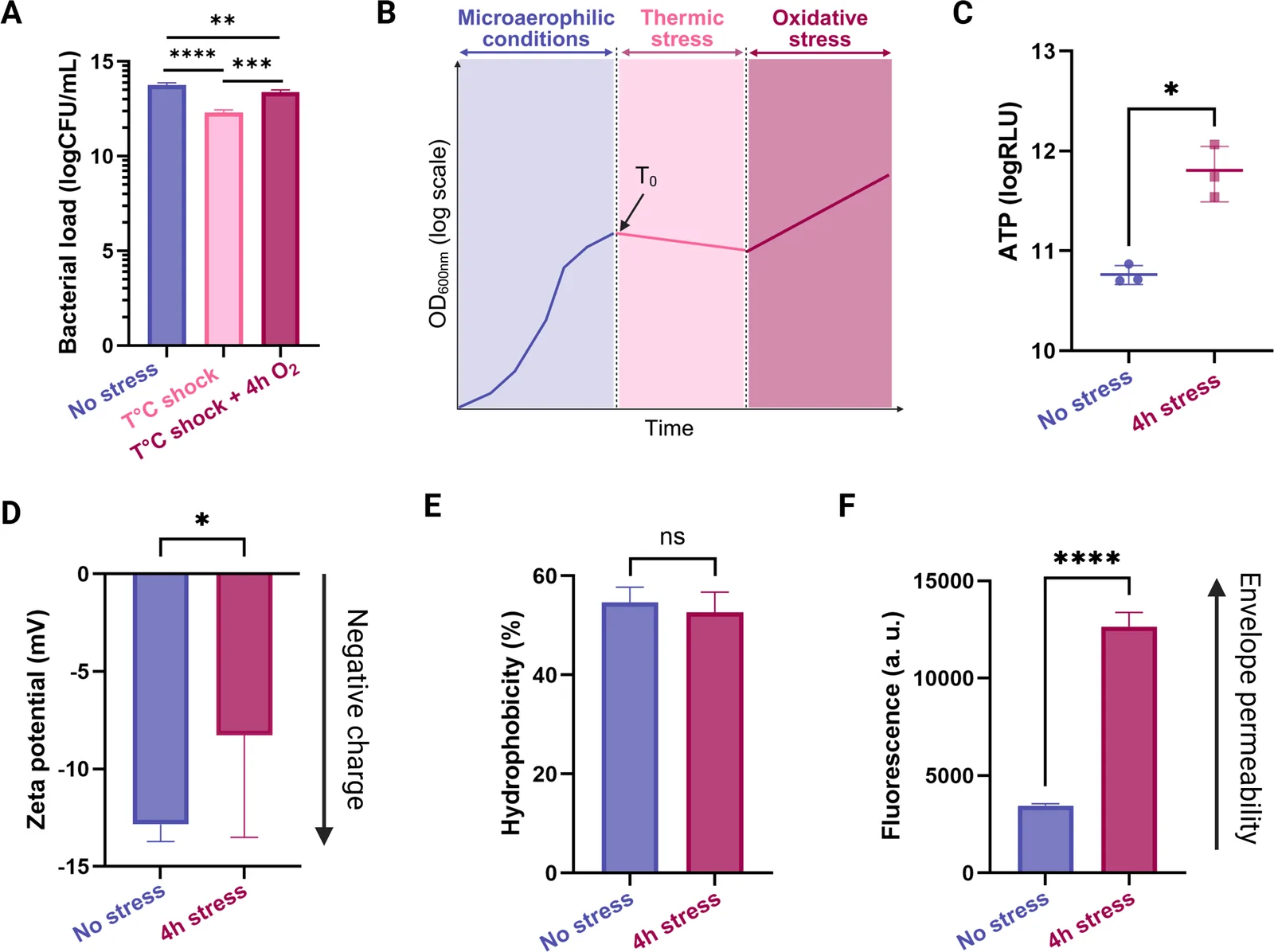
*C. jejuni* Bf multiplies under combined thermal and oxidative stress. (**A**) Bacterial load of *C. jejuni* in CFU/mL enumerated on plates before stress, after thermal shock, and after combined thermal and oxidative stresses. (**B**) Schematic illustration of a growth curve for *C. jejuni* Bf surviving combined thermal and oxidative stress. (**C**) ATP content of *C. jejuni* non-stressed and stressed cells, quantified using a luciferase-based assay. (**D**) Zeta-potential measurements of electrophoretic mobility of bacteria reflecting their surface charge. (**E**) Cell surface hydrophobicity based on the affinity of cells for xylene solvent. (**F**) Bacterial cell wall permeability measured with the ethidium bromide (EtBr) influx assay. Data are presented as mean ± standard deviation from independent experiments (*n* = 3). Statistical significance was determined by Student’s *t*-test; ns means not-significant, *P < 0.05, ** *P* < 0.01, ***P < 0.001, ****P < 0.0001.

We focused on the potential growth transition of the *C. jejuni* Bf strain from microaerophilic to stress conditions. *Campylobacter* grew exponentially under microaerophilic conditions, and at time zero (T_0_), cultures were subjected to successive heat and cold thermal shocks, which caused a slight but significant reduction (1 log) in the bacterial load. Surprisingly, although exposed to oxygen, the Bf cells resumed growth (Fig. 3A and B). To confirm that conditions mimicking poultry slaughtering steps do not induce a dormant state in *C. jejuni* Bf cells, ATP levels were measured in untreated and stressed cells 4 h post-stress. ATP can be detected only in live cells, as it is quickly depleted upon cell death, whereas dormant cells show a reduction in intracellular ATP levels (37). ATP levels were higher in cells subjected to consecutive stress than in untreated cells (*P*<0.05, Fig. 3C), indicating increased metabolic activity.

To determine whether the physicochemical characteristics of the cell envelope changed during adaptation of *Campylobacter* to stress, we examined the bacterial cell surface. Cells subjected to consecutive stress exhibited a less negative surface charge than untreated cells, as determined by zeta-potential measurements (−12.9 ± 0.9 mV in control vs −8.3 ± 5.2 mV in stressed cells, Fig. 3D), while no difference in cellular hydrophobicity was observed (Fig. 3E). Notably, a decrease in *Campylobacter* membrane potential was previously observed during bacterial incubation in sterile water and upon entering the VBNC state (38). Additionally, the EtBr diffusion assay showed significantly increased membrane permeability in cells subjected to consecutive stress compared to control cells (*P* < 0.0001, Fig. 3F). Increased EtBr influx is a hallmark of stress and typically indicates that the cell loses its ability to maintain membrane integrity or active efflux. Therefore, we speculated that compromised membrane integrity increases the production of bEVs.

### Combined stress changes the characteristics of bEVs released by *C. jejuni* Bf

**Fig 4:**
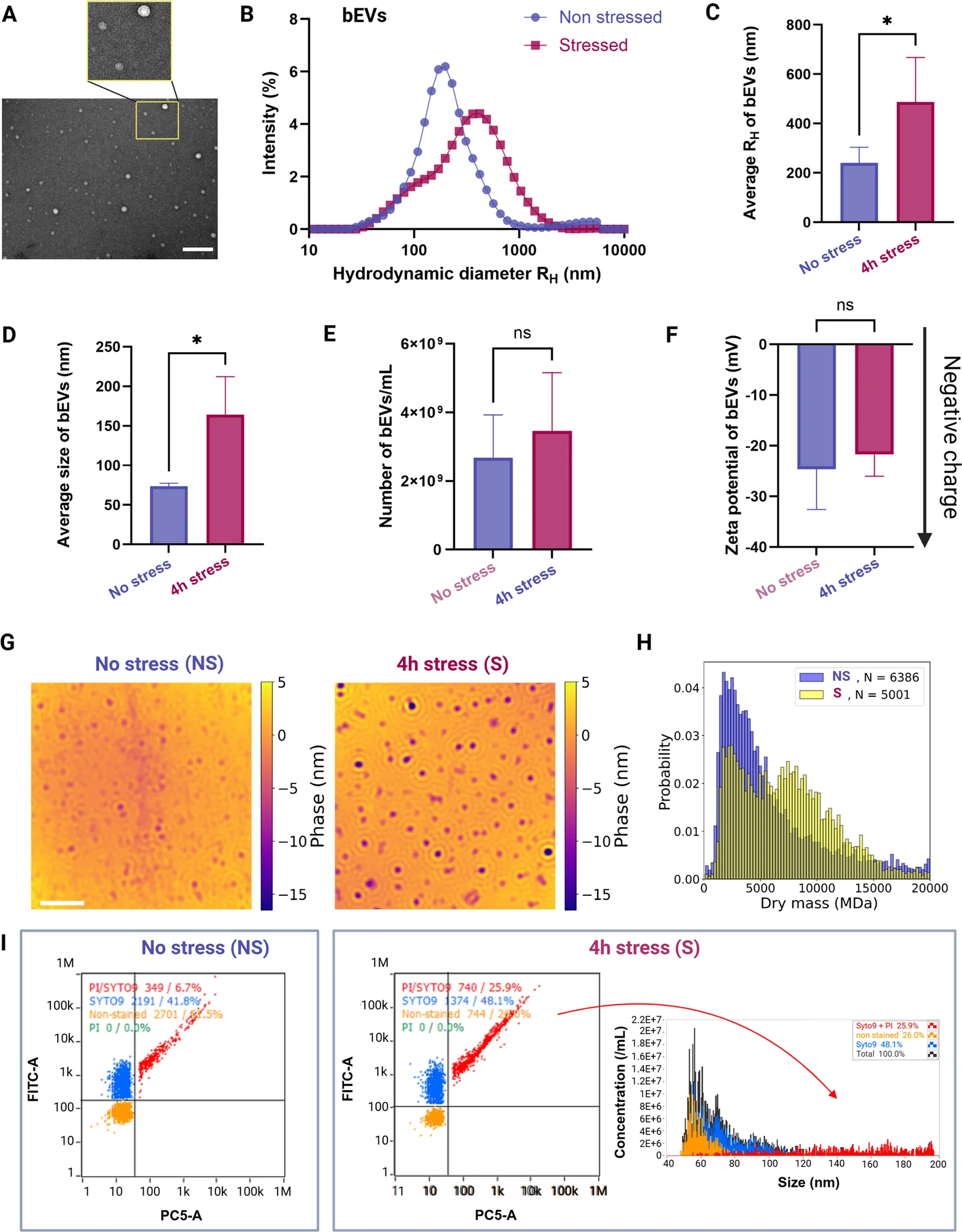
Under stress exposure*, C. jejuni* Bf cells produce larger and heavier extracellular vesicles carrying extracellular DNA. (**A**) Negative staining TEM micrographs of bEVs isolated from *C. jejuni* Bf culture. bEVs highlighted in the yellow square are shown enlarged above. The scale bar, 250 nm. (**B**) Hydrodynamic radius distribution of bEVs from non-stressed and stressed *C. jejuni* measured by DLS. (**C and D**) Average hydrodynamic radius and size of bEVs from the two groups obtained using DLS and NanoFCM, respectively. (**E**) Concentration of bEVs purified from a 60 mL culture of stressed and non-stressed *C. jejuni*, as determined by NTA. (**F**) Zeta-potential measurements reflecting the surface charge of bEVs secreted from stressed and unstressed *C. jejuni* Bf cells. (**G**) Quantitative phase images of bEVs from non-stressed and stressed *C. jejuni* imaged with Z-WIM. Scale bar, 5 µm. (**H**) Dry mass distribution of bEVs from the two groups measured using phase images. (**I**) DNA content of bEVs estimated using the Syto9/propidium iodide (PI) assay showing the presence of external (PI) or internal DNA (Syto9). The right panel illustrates that large vesicles from cells subjected to consecutive stress carried extracellular DNA. Data are presented as mean ± standard deviation from at least three independent experiments. Statistical significance was determined by student’s *t*-test. ns, not significant; *P < 0.05.

We isolated bEVs secreted by unstressed and stressed *C. jejuni* cells by ultracentrifuging the culture supernatants, followed by iodixanol-based density gradient centrifugation to remove protein aggregates, nonmembrane proteins, and membrane debris (Fig. S1). Consecutive fractions were examined by TEM, which showed that bEVs were distributed in fractions containing 10%–26% iodixanol (Fig. S1A and B). The bEVs isolated from *C. jejuni* appeared spherical with a clearly visible lipid bilayer (Fig. 4A). The hydrodynamic diameter (*R*_H_) of bEVs measured by DLS (dynamic light diffusion) showed that the density gradient purification step separated bEVs with *R*_H_ >80 nm from a population of smaller particles (*R*_H_ ~20 nm), which most likely corresponded to secreted proteins and their aggregates (Fig. S1C). Nanoflow cytometry of purified bEVs stained with MemGlow membrane dye confirmed the purity of the bEVs, with 84.8% intact vesicles and 15.2% open or collapsed vesicles (Fig. S1D).

The size distribution of bEVs, determined by DLS, showed that unstressed Bf cells produced a uniform population of vesicles, with a mean hydrodynamic diameter, *R*_H_, of 197.6 nm, while stressed bacteria released a heterogeneous population comprising subpopulations with median *R*_H1_ of 92 nm, and *R*_H2_ of 361 nm (Fig. 4B; Fig. S1B). Due to the generation of larger bEVs under stress conditions, the mean bEV size determined by DLS and nanoflow cytometry (nFCM) was higher for stressed (486.78 nm in DLS, 164.34 nm in nFCM) than for non-stressed bacteria (240.71 nm in DLS, 73.41 nm in nFCM) (Fig. 4C and D). These results indicate that stressed *C. jejuni* secrete bEVs approximately twice as large as those produced under microaerophilic conditions.

Across conditions, the mean vesicle yield in terms of particles per milliliter was similar between non-stressed and stressed samples with 2.6 × 10^9^ ± 1.25 × 10^9^ and 3.5 × 10^9^ ± 1.69 × 10^9^ particles/mL respectively, as determined by NTA (Fig. 4E). Despite their different size distributions, the surface charge of bEVs from non-stressed and stressed bacteria was comparable, with average values of −24.6 ± 8.0 mV and −21.7 ± 4.3 mV, respectively, as measured by zeta potential (Fig. 4F). Notably, compared to the bacterial cells themselves (mean surface charge of −10.5 mV; Fig. 3D), the bEVs exhibited a markedly more negative surface charge (mean of −23 mV), suggesting selective integration of anionic membrane components during vesicle formation.

To further verify whether, under combined stress, larger bEVs were produced to transport more biological molecules than the control, we estimated their dry mass using a quantitative phase imaging (QPI) technique called Z-WIM. The increased sensitivity of the Z-WIM method allows the precise weighing of the dry mass of individual vesicles by phase (39). As shown in the phase images, Z-WIM confirmed that bacterial cells subjected to consecutive stress produced larger vesicles than unstressed cells (Fig. 4G). In addition, larger bEVs were heavier than those secreted by non-stressed cells, reflecting differences in their cargo (Fig. 4H). The dry mass of bEVs produced by non-stressed and stressed bacteria averaged 6,063 MDa and 7,141 MDa, respectively (Fig. 4H). For comparison, the dry mass of empty DOPC liposomes with a diameter of 100 nm was approximately 120 MDa (40). These results show that after consecutive thermal and oxidative stress, *C. jejuni* produces bEVs in similar numbers and with similar surface charges as non-stressed cells, but with increased sizes, *R*_H_, and dry mass, potentially reflecting differences in their cargo.

We previously showed that *C. jejuni* under microaerophilic conditions secretes a heterogeneous population of bEVs, with approximately 42% carrying encapsulated DNA (17). Additionally, bacterial DNA and bEVs have been reported in human blood circulation (41). To determine whether cells subjected to consecutive stress also use bEVs to export DNA, we labeled bEVs with Syto9 and PI and performed nFCM to measure their DNA content. Because PI cannot permeate intact membranes, while Syto9 can, this dual staining allows discrimination between internal and external DNA. As expected, 41.8% of bEVs produced by non-stressed bacteria were stained only with Syto9, and 6.7% were double-stained with Syto9 and PI (Fig. 4I, left panel). In contrast, the population of bEVs secreted by stressed *C. jejuni* contained 48.1% Syto9-positive bEVs and 25.9% double-stained bEVs (Fig. 4I, right panel). No bEVs were stained with PI alone, indicating that none displayed surface-associated DNA without the presence of internal DNA. Moreover, DNA localization correlated with vesicle size under stress conditions: DNA was mainly detected inside bEVs of median diameter (Syto9-stained, blue population), whereas larger bEVs (≥80 nm) exhibited both internal and surface-associated DNA (Syto9/PI-stained, red population). In contrast, smaller bEVs (≤60 nm) contained no detectable DNA (unstained orange population in Fig. 4I). The presence of DNA in gram-negative bacterial bEVs is usually associated with explosive cell lysis. However, the secretion of larger bEVs containing chromosomal DNA is also associated with various abiotic stressors and/or biofilm formation (42).

### *C. jejuni* produces bEVs with a distinct lipid signature under stress conditions

**Fig 5:**
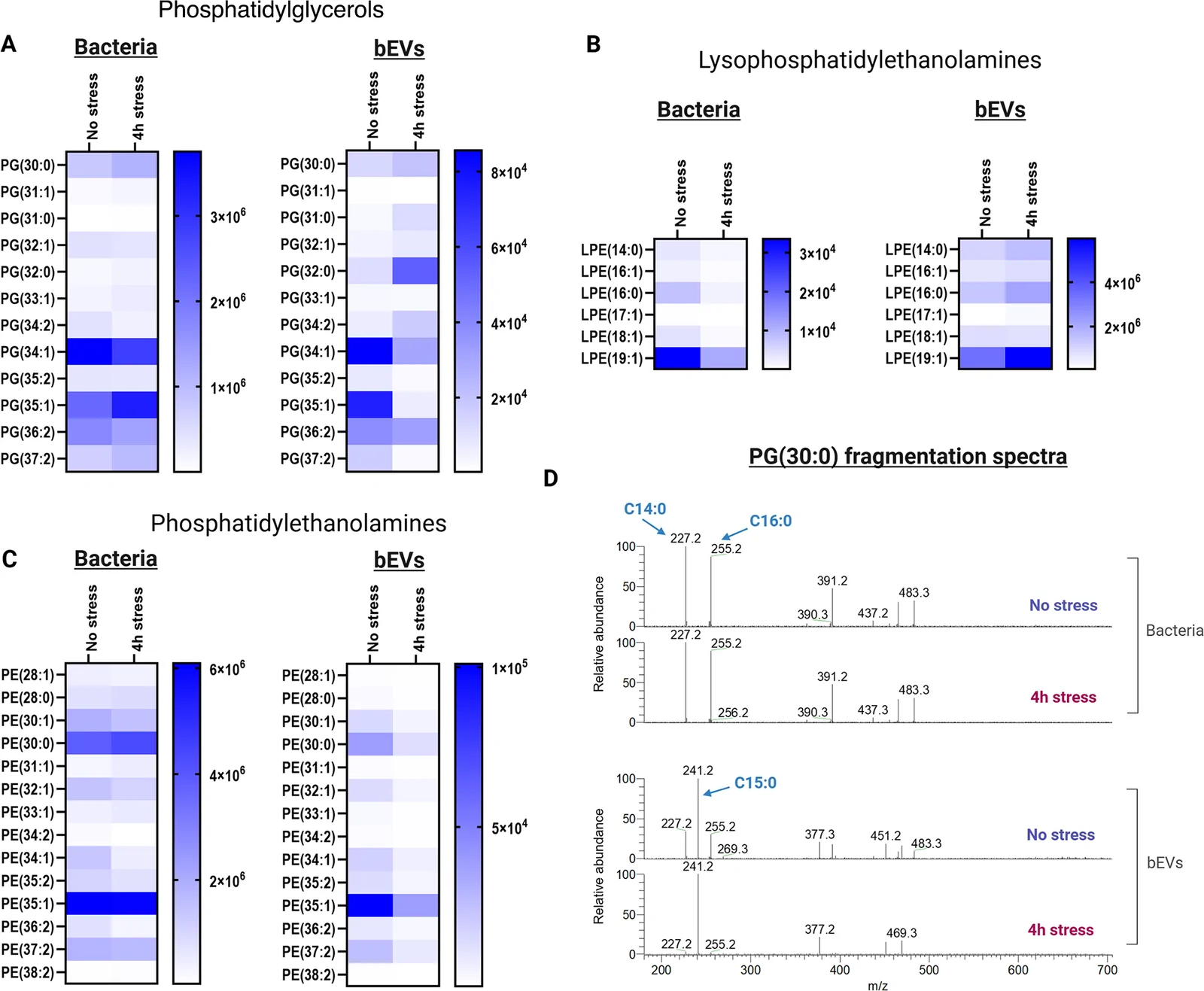
Stress exposure influences the lipid composition of cells and vesicles secreted by *C. jejuni* Bf. PLs were extracted from the bacterial pellets or purified bEVs and separated by polarity. Individual species were identified in negative electrospray ionization mode (ESI−) using high-resolution mass detection. (**A, B, and C**) Heatmaps showing the PG, LPE, and PE phospholipids identified in all samples, and the relative abundance of the constituent fatty acids (ranging from 0% in white to 100% in dark blue). (**D**) Mass spectral profiles of the species PG(30:0) across all samples. The x-axis shows the m/z ratio of the ions, and the y-axis indicates their relative abundance. Note significant differences in PG profiles between the plasma membrane and bEVs.

To further investigate membrane remodeling in *C. jejuni*, we performed exploratory untargeted lipidomic analysis by mass spectrometry on both bacterial cells and their bEVs, using a previously optimized chromatographic method for total lipid analysis (43). We identified 43 lipid species in both samples. All species belonged to three lipid classes: phosphatidylethanolamines (PE) (19 species), phosphatidylglycerols (PG) (18 species), and lysophosphatidylethanolamines (LPE) (6 species). Surprisingly, non-bilayer anionic cardiolipin species were not detected in *C. jejuni* Bf, although cardiolipin is abundant in most bacteria (44). For each phospholipid (PL) species, the absolute peak intensity was compared between conditions for both bacterial cells and bEVs (Table S1). The peak intensities of PG, PE, and LPE are represented as heat maps in Fig. 5A through C, respectively. To facilitate comparison between conditions, a fold change (FC) of >4 was considered significant.

In bacterial cells, no significant differences were observed in PG, PE, or LPE lipid species between the stressed and non-stressed conditions (Fig. 5A through C, left panels). In contrast, marked differences were detected in the lipid composition of bEVs derived from stressed and non-stressed cells. Within the PG class, the PG(31:0) species (corresponding to PG(14:0-17:0) and PG(15:0-16:0) in the bEV-stressed sample) and PG(32:0) species (corresponding mainly to PG(15:0-17:0) in both bEVs samples) were increased 5-fold and 4.7-fold, respectively, in stressed bEVs compared to non-stressed bEVs (Fig. 5A, right panel). The only other PG species containing odd-chain fatty acids (FAs; C15:0 and C17:0) and PG(30:0) are uniquely composed of PG(15:0-15:0) in stressed bEVs, whereas they are composed of both PG(15:0-15:0) and PG(16:0-14:0) in non-stressed bEVs. This suggests that stress induces the production of bEVs enriched in saturated odd-chain FAs in the PG lipid class in *C. jejuni*.

Interestingly, lipidomic analysis revealed that all PG species containing C19:1 were significantly reduced in the bEV membranes following stress. It should be noted that lipidomic analysis does not allow discrimination between unsaturated linear lysophospholipids and cyclopropane lysophospholipids containing 19 carbons. However, according to our FA analysis (next section), C19:1 likely corresponds to the cyclopropane C19. For several lipid species, this decrease was substantial in bEVs following stress, including an 11-fold reduction in PG(35:1) (corresponding to PG(16:0-19:1)) and an 8-fold reduction in PG(37:2) (corresponding to PG(18:1-19:1)) (Table S1 and Fig. 5A).

The abundance of LPE(16:0) and LPE(18:1) was reduced after stress in bEVs membranes, with 4.7-fold and 4.9-fold decreases, respectively (Fig. 5B). Other LPE species (LPE(14:0), LPE(16:1), LPE(17:1), and LPE(19:1)) were present at comparable levels in bEVs under the two conditions. Finally, no significant differences were observed within the PE lipid classes between conditions (Fig. 5C, right panel).

The most striking difference was observed in the PG composition between bacterial cells and vesicles under both conditions. Identical PG MS1 m/z displayed distinct FA compositions in the cellular and vesicular membranes, with bEVs being enriched in PG species containing saturated odd-chain FAs such as C15:0 and C17:0, which were not detected in *C. jejuni* cell membranes. As illustrated in Fig. 5D, PG(30:0) was only composed of PG(14:0-16:0) in the bacterial membrane, whereas it mainly consisted of PG(15:0-15:0) in the bEVs (Fig. 5B). Similarly, PG(32:0) consisted largely of PG(16:0-16:0) in the stressed bacterial membrane but was mainly composed of PG(15:0-17:0) in bEVs, suggesting specific remodeling of PG species during vesicle biogenesis.

### Combined stress drives unsaturated FA accumulation in *C. jejuni* and cyclopropane FA depletion in bEVs

**Fig 6:**
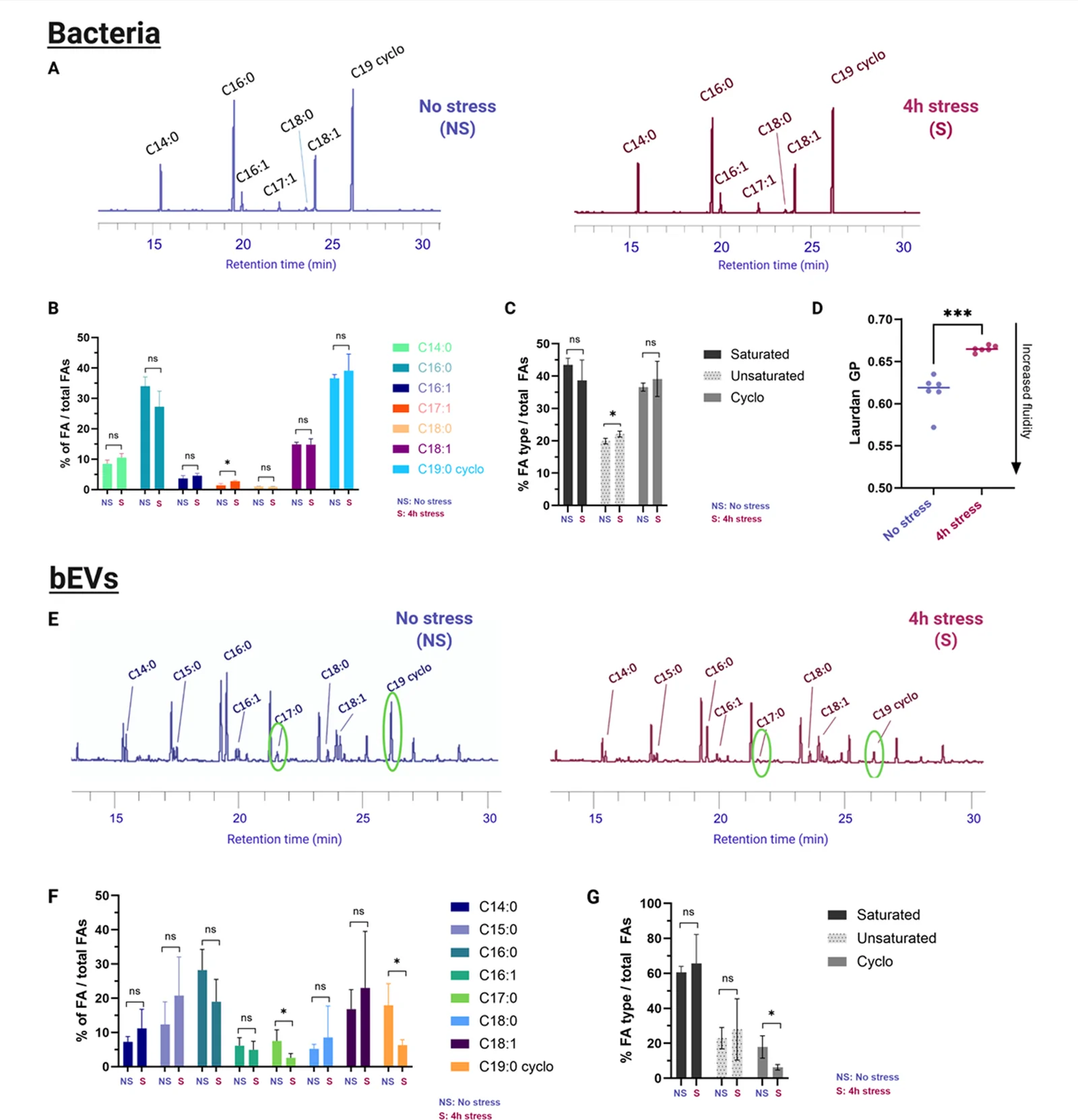
Fatty acid compositions of *C. jejuni* Bf cell and vesicle membranes change under stress exposure. (**A**) Gas chromatograms of fatty acids composing the membranes of stressed and non-stressed *C. jejuni* cells. (**B**) Relative abundance of detected fatty acids from the cell membrane, in %. (**C**) Relative abundance of fatty acid groups (saturated, unsaturated, cyclo-FA) in the membranes of stressed and non-stressed cells, in %. (**D**) Membrane fluidity measurements using Laurdan generalized polarization (GP) of stressed and non-stressed *C. jejuni* cells. Increasing relative Laurdan GP indicates membrane rigidification upon stress exposure. (**E**) Gas chromatograms of fatty acids composing the membranes of bEVs originating from stressed and non-stressed *C. jejuni* cells. (**F**) Relative abundance of all fatty acids composing the vesicle membrane, in %. (**G**) Relative abundance of fatty acid groups (saturated, unsaturated, and cyclo-FA) in the membranes of vesicles released by stressed and non-stressed cells, in %. Peaks corresponding to fatty acids significantly less abundant in stressed vesicles are circled in light violet. Student’s *t*-test was used to calculate *P*-values. ns, not significant, *P < 0.05, *** *P* < 0.001.

Next, we quantified the FAs in *C. jejuni* Bf cellular and vesicle membranes. In both non-stressed and stressed cells, the bacterial cells contained seven main FAs: C14:0, C16:0, C16:1, C17:1, C18:0, C18:1, and C19 cyclo (Fig. 6A). This FA composition is consistent with the previously reported representative FA profile of most *C. jejuni* strains (45), except for hydroxylated FAs such as 3-OH-C14:0, which were not detected. To date, limited information is available regarding the membrane composition of *C. jejuni*. Evidence suggests that its membrane is mainly composed of straight-chain fatty acids rather than branched ones and contains nine phospholipid classes, with lysophospholipids accounting for a substantial fraction (30%–45%) of the total phospholipid content (46). Lysophospholipids are formed as intermediates in phospholipid synthesis or as metabolites of phospholipid degradation (47, 48).

To further verify the absence of hydroxylated FAs in the *C. jejuni* Bf strain, we extracted FA methyl esters using diethyl/cyclohexane instead of diethyl/heptane (49). However, this approach, which allows the extraction of hydroxylated FAs, produced FA profiles comparable to those obtained using classical extraction (Fig. S2), further suggesting that Bf did not produce hydroxylated FAs under the tested conditions. The FA analysis showed that cells subjected to consecutive stress had a significantly higher proportion of C17:1 compared to controls (2.76% vs 0.68%, respectively; Table S1 and Fig. 6B), resulting in an overall increase in unsaturated FA content (Fig. 6C). No significant differences were detected in the levels of saturated and cyclopropane fatty acids.

Modification of the membrane composition may preprogram membrane fluidity to maintain its function. Therefore, we measured the fluidity of *C. jejuni* Bf before and after consecutive stresses by measuring Laurdan GP. The Laurdan probe intercalates into the lipid bilayer and displays an emission wavelength shift depending on the number of water molecules in the membrane (50). Accordingly, an increase in the proportion of unsaturated FAs in the membrane leads to increased fluidity because the presence of double bonds in *cis* configuration introduces kinks in the carbon chain. Surprisingly, under combined oxidative and thermal stress, the diderm of *C. jejuni* cells was significantly more rigid than that of non-stressed cells (Fig. 6D).

Eight FA species were identified at the vesicle level: C14:0, C15:0, C16:0, C16:1, C17:0, C18:0, C18:1, and C19 cyclo (Fig. 6E and G). Notably, C15:0 was detected in bEVs but not in the plasma membrane. Moreover, the FA profiles of bEVs differed significantly from those of their parental cells, with bEVs displaying many unidentified peaks, often immediately before the major fatty acid peak (Fig. 6A and E). These unidentified peaks could be modified FAs, likely differing at the branching level. For example, just before C16:0, there could be C16:0 with a different branching pattern at the end of the chain, causing it to appear slightly later. This hypothesis needs to be confirmed by including standards for branched FAs from *Campylobacter* or a closely related bacterium.

The relative abundances of C17:0 and C19 cyclo were significantly lower in bEVs from cells subjected to consecutive stress than in bEVs from non-stressed cells (Fig. 6E and G). This finding, along with the specific lipid profiles (Fig. 5C), shows that consecutive thermal and oxidative stress accumulates unsaturated odd-chain FAs in the *C. jejuni* cell membrane. As odd-chain FAs alter lipid packing differently than even-chain FAs, our results suggest that C17:0 plays a key role in fine-tuning membrane fluidity and helping *C. jejuni* survive temperature changes and oxygen, which leads to the generation of bEVs with lower levels of C17:0 and C19 cyclo.

### Stress impacts protein content in *C. jejuni* cells and their bEVs

We then performed a comparative nano-LC-MS/MS label-free analysis and identified 660 proteins in control cells and 424 proteins in cells subjected to consecutive stress (4 h post-stress) (Table S2). The relatively low number of identified proteins is due to the limited reviewed annotation of the *Campylobacter* proteome in the UniProt database (see Section Proteomic studies).

**Fig 7:**
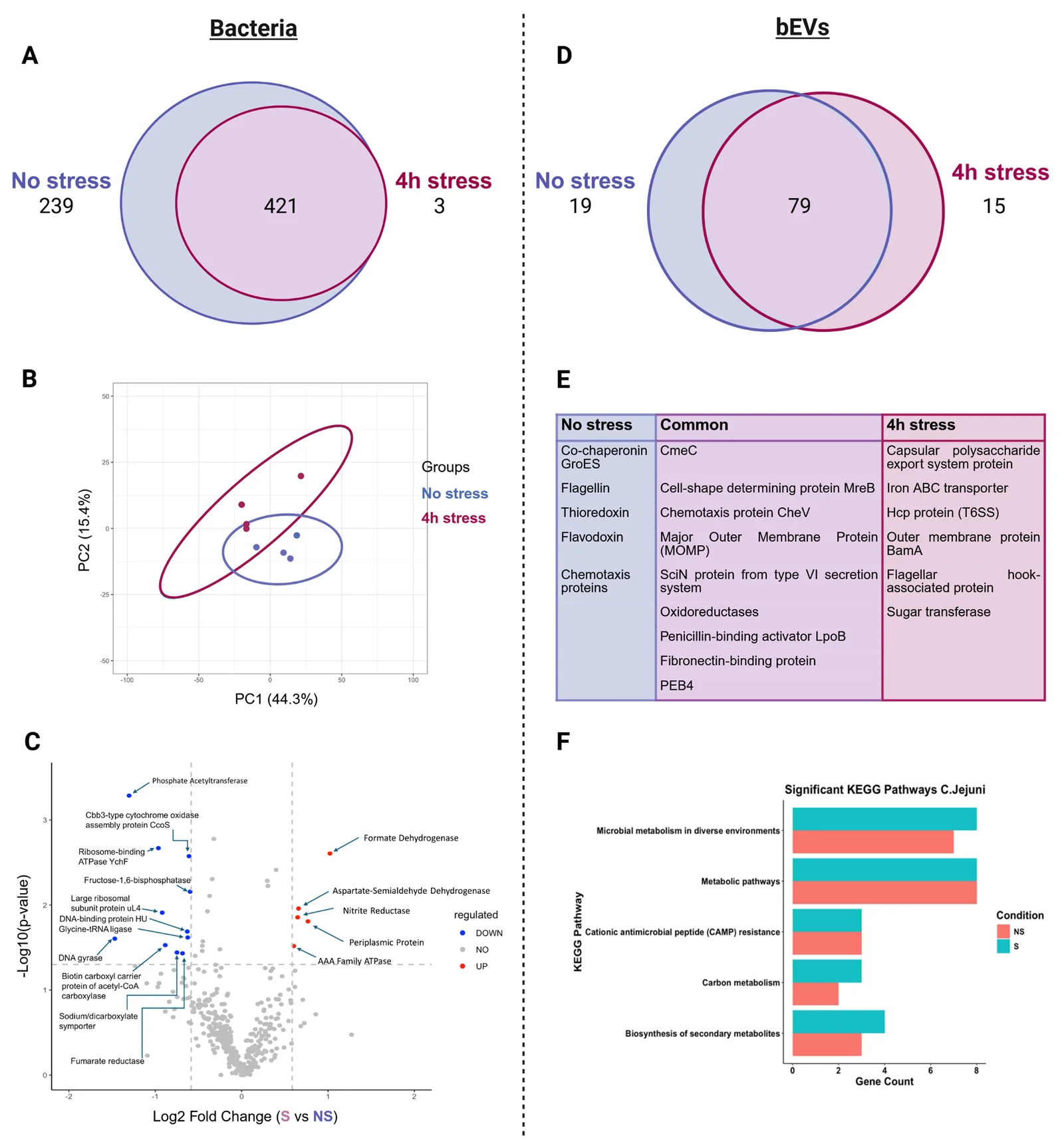
Stress exposure leads to protein content modifications in *C. jejuni* Bf. (**A**) Venn diagram shows the number of proteins identified in stressed and non-stressed *C. jejuni* cells. (**B**) PCA plot based on scaled LFQ intensities for identified proteins in stressed and non-stressed cells. Ellipses indicate the 95% confidence interval for each group. (**C**) Volcano plot illustrates differentially expressed proteins in cells subjected to consecutive stress compared to controls (non-stressed). The x-axis shows the log2 fold change, and the y-axis shows −log10 (*P*-value). The significance threshold is set at adjusted *P*-value < 0.05. (**D**) Venn diagram shows the number of proteins identified in bEVs from both stressed and non-stressed *C. jejuni*. (**E**) Table listing the major proteins found only in one bEV group (stressed or non-stressed) or in both bEV groups. (**F**) Bar plot showing significant KEGG pathways, with a *P*-value threshold of 0.05. Data were obtained from independent experiments (biological replicates *n* = 5 for bEVs, *n* = 4 for cells).

A principal component analysis (PCA) based on imputed label-free quantification (LFQ) intensities, requiring proteins to be present in at least 50% of the samples in one group, showed overall variance between the non-stressed and stressed samples, with some slight overlap between the two ellipses (Fig. 7A). We speculate that this small overlap reflects the non-simultaneous cell morphological changes observed in Fig. 2A and B. Hierarchical clustering (HC) analysis revealed significant variations in protein profiles between conditions but not substantially between sample replicates (Fig. S3). The Venn diagram of the identified proteins showed that non-stressed and stressed *C. jejuni* cells contained 239 and three unique proteins, respectively, and more than 63% (421 out of 663) of the identified proteins were present in both (Fig. 7B; Table S2). Notably, this analysis indicates that 36% of the proteins (239 out of 663) identified in the control cells were not found in cells subjected to consecutive stress. Among the uniquely expressed proteins in stressed cells, we found NAD^+^-dependent protein deacylase, which removes acyl groups from lysine residues in *Campylobacter* (51). This enzyme may contribute to the reduced negative bacterial charge observed in the zeta-potential experiments (Fig. 3D).

In addition to the differentially detected proteins, five proteins were enriched, and 11 proteins were downregulated in the stress category compared to the controls (using a *P*-value threshold of <0.05, and an FC threshold of >1.5 or <0.67) (Fig. 7C). Upregulated proteins are involved in energy metabolism, including formate dehydrogenase (which catalyzes electron transfer via menaquinone and contributes to ATP synthesis) (52), and nitrate reductase (which transfers electrons from menaquinone to nitrite, supporting ATP synthesis) (53), which may explain the high levels of ATP present in cells subjected to consecutive stress (Fig. 3C). The aspartate semialdehyde dehydrogenase, periplasmic protein, and AAA family ATPases were also upregulated. These proteins are linked to cell growth and metabolism, confirming that, at the early stage of *C. jejuni* adaptation to oxidative and thermal stress, bacterial cells still undergo biosynthesis and protein remodeling. In particular, periplasmic proteins play a critical role in supporting *Campylobacter* survival in environmental reservoirs, including water and food matrices (54). The downregulation of key enzymes of anaerobic respiration, cbb3-type cytochrome C, which has high oxygen-binding affinity (55), and fumarate reductase of the succinate:quinone oxidoreductase family (56), reflects the adaptation of the bacterium to oxidative stress associated with the general metabolic shift in dormant states.

Protein profiling of bEVs secreted by stressed and non-stressed *C. jejuni* cells identified 113 protein groups in 5 biological replicates for each condition (Table S2). Protein distribution was balanced among the samples, with 70% (79 out of 113) of protein groups shared between both conditions, and 19 and 15 unique proteins found in bEVs from unstressed and cells subjected to consecutive stress, respectively (Fig. 7D). The *Campylobacter* vesicle marker MOMP (15, 17) was detected in all the tested samples (Fig. 7E). *C. jejuni* Bf does not have a gene encoding a cytolethal distending toxin, but most unique proteins in bEVs of stressed cells are virulence factors, including the iron ABC transporter (57), capsular polysaccharide export system proteins (58), flagellar hook-associated protein (59), BamA, and Hcp (15). In contrast, unique proteins in bEVs from control cells were involved in central metabolism and protein folding, such as the oxidoreductase group (60), thioredoxin, GroES (59), and chemotaxis proteins (61), as summarized in Fig. 7E. KEGG (Kyoto Encyclopedia of Genes and Genomes) pathway analysis confirmed cell metabolic adaptations to stress, with differentially expressed proteins involved in metabolic and biosynthetic interactions (Fig. 7F). While these results provide valuable insights, they should be interpreted with caution given the limitations of the data set, including the limited number of proteins identified.

Our proteomic analysis suggests that general stress responses and metabolic changes occur as *C. jejuni* copes with consecutive thermal and oxidative stress. In addition, consecutive stress alters the bEVs cargo.

### Stress enhances the cytotoxicity of both *C. jejuni* cells and bEVs toward Caco-2 cells

Considering the changes observed in bacterial cells subjected to consecutive stress compared to non-stressed bacteria, we investigated the effect of two groups of *C. jejuni* on Caco-2 tight junctions and the possible involvement of bEVs in disrupting the epithelial barrier.

**Fig 8:**
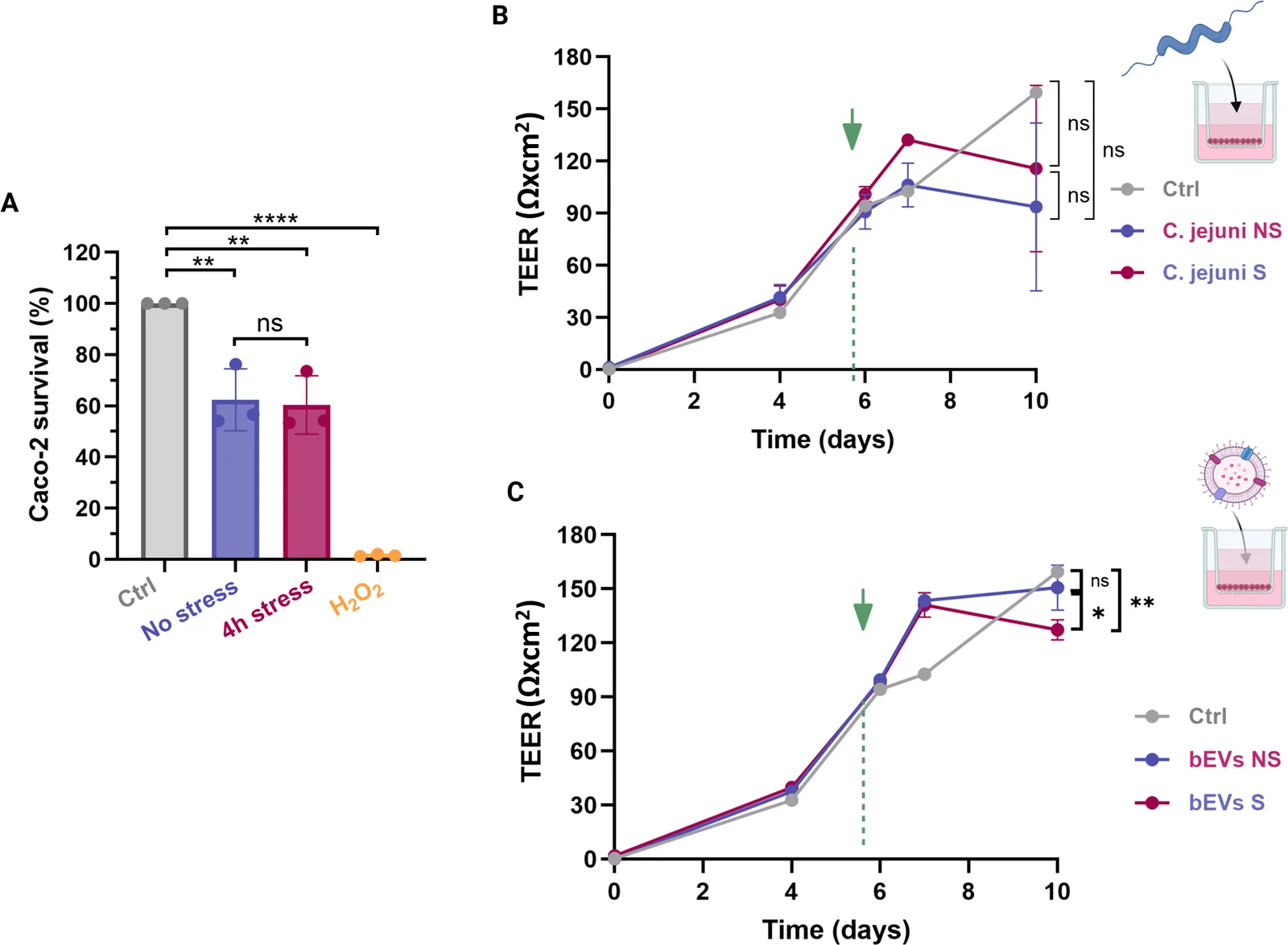
Exposure to stress increases the cytotoxicity of *C. jejuni* vesicles toward Caco-2 cells. (**A**) The toxicity of *C. jejuni* purified bEVs was evaluated by the MTT test on a confluent monolayer of Caco-2 cells at an MOI of 20. TEER (transepithelial electrical resistance) measurements of the Caco-2 cell monolayer were taken after challenge with either *C. jejuni* Bf cells (**B**) or purified bEVs (**C**) added to the apical channel. TEER experiments were performed on day 6 when Caco-2 confluence was reached, as indicated by a green arrow, with bEVs at an MOI of 20. Error bars indicate the standard deviation from three biological replicates. Student’s *t*-test was used to calculate *P*-values. Data are presented as mean ± standard deviation from independent experiments (*n* = 3). ns, not significant, *P < 0.05, **P < 0.01, ****P < 0.0001.

We first evaluated the cytotoxic effect of *C. jejuni* culture supernatant at 20% (containing diluted bEVs) and purified bEVs at an MOI of 20 on Caco-2 cells in plates using the MTT viability assay (62). While supernatants showed no reduction in cell viability, bEVs purified from non-stressed and stressed *C. jejuni* killed approximately 38% and 40% of treated Caco-2 cells within 24 h (Fig. 8A). This decrease was not statistically significant between bEVs from non-stressed and stressed bacterial cells.

We evaluated Caco-2 tight junctions using a quantitative TEER assay, which assesses epithelial barrier integrity (17). Caco-2 cells were cultured to reach TEER values exceeding 100 Ω/cm², indicating formation of intact tight junctions and low paracellular permeability. Cell confluence was confirmed by optical microscopy (Fig. S4). Stressed and non-stressed bacteria were then introduced into either the apical channel (Fig. 8B) or the basal channel (Fig. S5). In the control wells, TEER values continued to increase, indicating ongoing cell growth through the end of the experiment. However, after bacterial inoculation in the basal channel, Caco-2 cells continued to grow for 1 day, after which the TEER value began to decrease from 134.7 ± 8.3 to 86.3 ± 20.4 Ω/cm² for non-stressed *C. jejuni* and from 148.9 ± 46.1 to 46.1 ± 32.4 Ω/cm² for stressed *C. jejuni*, indicating that *C. jejuni* compromised the integrity of tight junctions in the Caco-2 monolayer. No significant difference was observed between the toxicity of stressed and non-stressed bacteria (*P* > 0.05) (Fig. S5; Fig. 8B and Table S3).

Next, we examined the effect of bEVs released by stressed and non-stressed *C. jejuni* on Caco-2 tight junctions using TEER experiments. After bEV addition, cells continued to proliferate for 1 day, but then the TEER value in wells treated with stressed bEVs decreased from 141 ± 6.8 to 127 ± 5.5 Ω/cm², whereas in wells treated with bEVs from non-stressed *C. jejuni*, the TEER values slightly increased from 141 ± 0.69 to 150 ± 12.5 Ω/cm² (Fig. 8C and Table S3). This suggests that bEVs from stressed *C. jejuni* cells disrupt more tight junctions in Caco-2 monolayers than bEVs from non-stressed cells, which can facilitate bacterial infection of the host.

## DISCUSSION

In this study, we provide evidence that aerotolerant *C. jejuni* Bf can survive and multiply under combined thermal and oxygen stress. During the early stage of adaptation (4 h post-stress), the bacterial cells exhibited lipidomic, proteomic, and morphological responses and released bEVs with increased toxicity to human epithelial barrier integrity.

*C. jejuni* is an enterohepatic species that inhabits the intestinal tract or gall bladder of its hosts. It commonly exists as a commensal bacterium in a wide range of animals, and its transmission to humans via food and water strongly depends on its strain aerotolerance. Aerotolerance is one of the main strategies employed by bacteria to survive harsh stress conditions, along with biofilm formation and the transition to the VBNC state (63, 64). However, the aerotolerant state of *Campylobacter* remains largely uncharacterized. Studying combined stresses can predict *C. jejuni* behavior during poultry processing, as cell history plays an important role in adaptation by inducing physiological responses involved in co-selection mechanisms (2, 25). *Campylobacter* strains that persist under harsh environmental conditions would provide a reservoir for human infection.

Multilocus sequence typing (MLST) of *C. jejuni* strains showed that aerotolerant strains were primarily classified into MLST clonal complexes CC-21 and CC-45, which are also major complexes implicated in human gastroenteritis (20, 65). In contrast, *C. jejuni* strain Bf studied here belongs to the less common clonal complex ST-403, whose strains have been recovered from patients with gastroenteritis and Guillain–Barré syndrome (34, 66, 67). The Bf strain is known as an atypical aerotolerant strain and can be considered hyper-aerotolerant because it survives for more than 24 h exposed to ambient atmospheric conditions (Fig. 2A [34]).

After combined thermal and oxygen stress, the Bf strain showed a significant decrease in motility and remodeled its membrane, allowing a subset of the cell population to change from S-shaped to coccoidal (Fig. 1; Videos S1 and S2). The strain was relatively robust, as not all cells adopted the coccoidal shape; some remained S-shaped and survived (Fig. 2A). These results may indicate heterogeneous adaptation within the population after combined stress, with multiple morphologies conferring resistance, as observed in some other bacteria (68). Although it was previously shown that Bf can enter the VBNC state in its spiral form (35), our results suggest that under combined stress, Bf cells did not enter the VBNC state or did so only partially. In fact, the intracellular ATP level remained high and the cells continued to multiply slowly (Fig. 3A and C), indicating that at least part of the population is not dormant. While many stress conditions decrease ATP due to growth inhibition or metabolic slowdown, several studies indicate that transient or mild stress conditions can induce short-term increases or stabilization of ATP levels associated with adaptive metabolic responses and subsequent behavior regarding further stresses (19, 26, 27). In line with this, our proteomic analysis indicated that proteins involved in energy metabolism, cell metabolism, and growth were upregulated following combined stress (Fig. 7C), enabling cells to multiply under harsh conditions.

We also showed that aerotolerance fine-tunes the physicochemical properties of the cell envelope, which tend to be more rigid and mechanically resistant. Notably, the cell surface became less negatively charged (Fig. 3D), suggesting that the consecutive stresses modified the expression of surface-charged molecules such as lipooligosaccharides, proteins, and lipids. The S-to-coccoid transition in *C. jejuni* was previously shown to involve enzymatic remodeling and compaction of the peptidoglycan layer but not extrusion or loss of the cell wall (69). In this regard, we found that cells subjected to consecutive stress showed increased envelope permeability (Fig. 3F), which likely reflects modifications at the membrane level.

The major finding of this study regarding *C. jejuni* membrane adaptation to combined stresses is that, while maintaining the lipid composition of its plasma membrane, the bacterium can modulate the lipid composition of secreted bEVs, which depends greatly on environmental conditions. The greater negative charge observed for *C. jejuni* bEVs compared to that of the bacterial plasma membrane (Fig. 4F vs Fig. 3D) may result from the selective enrichment of anionic lipid components during vesicle formation. This is consistent with previous findings that bEVs in gram-negative bacteria often originate from regions enriched in negatively charged lipids such as PG (70, 71). These lipids preferentially accumulate in curved membrane domains that favor vesicle budding. Functionally, a more negative surface charge can influence vesicle stability, interaction with divalent cations, or binding to host cell membranes, potentially modulating the biological activity of stress-derived bEVs.

Quantification of FAs in *C. jejuni* cell membranes indicated that the odd-chain unsaturated fatty acid C17:1 accumulated in the plasma membrane following stress, where it may have adopted a *trans* configuration to increase envelope rigidity. Combined thermal and oxidative stresses may explain the paradoxical increase in membrane rigidity, despite a higher proportion of unsaturated FAs (Fig. 6C). Although heat stress generally induces bacteria to increase saturated or longer-chain lipids to counteract the fluidizing effect of temperature (72–74), oxidative stress can promote lipid peroxidation and alter the configuration of double bonds (e.g., conversion from *cis* to *trans*). *Trans* unsaturation packs more tightly than *cis* unsaturation, thereby reducing the membrane fluidity. The interplay between these two stresses could lead to a lipid profile that maintains or even increases membrane rigidity, despite an apparent enrichment in unsaturated FAs. Simultaneously, the packaging of odd-chain fatty acids C17:0 and C19 cyclo was limited in bEVs following stress, suggesting their importance in maintaining the appropriate membrane FA composition of the parental cells. Selective incorporation of FAs in bEVs was also observed in *Helicobacter pylori*, with an increase in C17:0 and a substantial reduction in C14:0, C18:1, and C19 cyclo in bEVs under antibiotic pressure (75). Moreover, our findings indicate the contribution of lysophospholipids, which contain only one FA instead of two, to coping with stress. LPEs are virulence factors of *C. jejuni* that are required for bacterial motility under low-oxygen conditions (46, 76). In particular, short-chain LPE(14:0) is toxic to eukaryotic epithelial cells (76). Our lipidomic analysis revealed that LPE(14:0) was only slightly increased following stress in bEVs, but the abundance of LPE(16:0) and LPE(19:1) was highly reduced in the bEVs of stressed cells compared to that in non-stressed cells (Fig. 5C). Maintaining LPEs in the bacterial plasma membrane has detrimental effects on membrane integrity, as previously shown for *Escherichia coli* survival under stress conditions (77).

Overall, our results strongly suggest that bEVs released from cells subjected to consecutive stress are more likely produced through a programmed process rather than resulting from cell lysis. First, the lipid composition of bEVs differs from that of whole cells under both control and stressed conditions (Fig. 5). Second, cell counts and microscopic observations indicated that combined stress induced morphological changes but caused only a slight decrease in bacterial load (Fig. 2 and 3). Moreover, after 4 h post-stress, bacterial cells began to multiply (Fig. 3A). Fourth, the ATP level in the bacterial population increased in cells subjected to consecutive stress, directly indicating cell viability (Fig. 3C). Fifth, the number of bEVs in control and stressed populations is similar (Fig. 4E). Finally, proteomic data also suggest that bEV content is more likely programmed than a result of cell lysis (Table S2). For example, DnaK appears slightly more abundant in vesicles from stressed bacteria, while DnaJ appears less abundant. Since these two chaperone proteins are functionally closely linked and generally co-regulated, this result suggests a certain level of selection for the proteins incorporated into the vesicles rather than simply reflecting their intracellular abundance.

The ability of *C. jejuni* to cross-polarize epithelial cells and invade them is considered a hallmark of *C. jejuni* pathogenesis and has been extensively studied (17, 78–80). In this study, we found that both stressed and control *C. jejuni* cells could disrupt the epithelial barrier of Caco-2 cells (Fig. S4). However, the bEVs secreted following stress disrupted tight junctions in the Caco-2 monolayer more efficiently than the control bEVs (Fig. 8C). The disruption of junctional complexes in epithelial cells, which form a barrier to prevent microbes from crossing deeper tissues, is a key virulence strategy for *C. jejuni* and other diarrheagenic bacteria (15, 81). *C. jejuni* disrupts epithelial junctions mainly through the HtrA-mediated cleavage of adherens junctions, opening the paracellular pathway for bacterial internalization (15). However, HtrA was not differentially expressed between stressed and non-stressed cells, as determined by proteomic analysis to explain this finding (Table S2). Our results suggested that invasion factors transported by bEVs, such as flagella hook-associated protein and BamA, detected only in bEVs from cells subjected to consecutive stress (Fig. 7E), could facilitate bacterial penetration of the epithelium. This suggests that, under thermal and oxidative stress conditions, *C. jejuni* releases bEVs that may contribute to increased host colonization and infection. Therefore, based on our results obtained using the *C. jejuni* Bf strain, we propose that aerotolerant *Campylobacter* could employ a membrane fitness strategy to rapidly overcome stressful conditions and increase their virulence.

## MATERIALS AND METHODS

### Bacterial strains and culture conditions

*Campylobacter jejuni* strain Bf (35, 82) was stored at −80°C in brain heart infusion (BHI, Biomérieux, France) supplemented with 20% (vol/vol) glycerol. The strain was cultured on Columbia agar plates supplemented with 3% defibrinated horse blood for 24–48 h at 37°C under microaerobic conditions. Then, a liquid culture was prepared from a single colony of the plate in BHI broth (Biomérieux) supplemented with 50 µmol/L hemin (Sigma-Aldrich) and incubated for 18 h at 37°C under microaerobic atmosphere with shaking.

### Stress introduction protocol

*C. jejuni* Bf was subjected to consecutive stresses, inspired by the conditions encountered during poultry slaughter. First, 10 mL of the initial culture was immersed in a hot water bath (50°C) for 5 min and placed in a freezer (−20°C) for 4 min to induce thermal shock. Then, oxygen was introduced into the culture using a 10 mL syringe, and the culture tube was placed at 4°C with the cap open (to pursue oxygen exposure) for different times depending on the experiment.

### Optical microscopy for bacterial visualization

Optical microscopy was performed to visualize the bacterial cells exposed to the stress protocol. Observations were made with an Axio-Observer Z1 inverted microscope (Zeiss, Oberkohen, Germany) equipped with a Zeiss AxioCam MRm digital camera using a 100× oil immersion objective lens. Images were acquired using the ZEN software package (Zeiss, Oberkohen, Germany) and analyzed using the Fiji software (83) (https://imagej.net/ij/).

### Bacterial viability assay

To assess viability after thermal and oxidative stress, *C. jejuni* Bf was first cultured for 16 h at 37°C under microaerophilic conditions and then subjected to the stress protocol described before. The viable population was quantified using the plate enumeration method. Briefly, 10-fold serial dilutions of the bacterial suspensions were made before stress, after thermal shock, and after 4 h of air exposure, and 100 µL of each suspension was spread on BHI supplemented with 1% horse blood. All experiments were performed in triplicate with two plates/dilutions to allow statistical conclusions.

### Intracellular ATP quantification

The intracellular ATP content of the bacteria was determined using a luciferase-based BacTiter-Glo Microbial Cell Viability Assay (Promega, #G8230). As per the manufacturer’s instructions, 100 µL of each culture was mixed in a black, clear-bottomed 96-well plate (Greiner Bio-One, 655090) with 100 µL of room-temperature-equilibrated BacTiter-Glo reagent and incubated at 25°C in the dark with shaking (160 rpm) for 5 min. Relative luminescence units (RLU) were recorded using a TECAN Spark multimode microplate reader (Tecan Life Sciences, Switzerland). Fluorescence values were normalized to OD_600_ = 1 to allow for comparison between samples.

### Electrophoretic motility and hydrophobicity

The electrophoretic motility of *C. jejuni* cells and bEVs was analyzed by zeta potential measurements using a Zetasizer Pro (Malvern Panalytical Instrumentation, UK) and disposable capillary cells DTS1070. At least three measurements were performed for each sample, and the results are presented as the mean ± SD. The hydrophobicity of the *C. jejuni* cell surface was assessed using the method of microbial adhesion to solvents by comparing the cell affinity to the polar (PBS) and the non-polar solvent (xylene), as previously described (84) with some modifications. Xylene (300 µL) was added to the test tubes containing 2 mL of washed bacterial cells (~10^8^ CFU/mL) in PBS. After a 10-min incubation at 30°C, upon vortexing for 120 s, the suspensions were allowed to separate into the polar and non-polar phases. The polar phase of PBS was carefully removed with a Pasteur pipette and transferred to a UV-Vis cuvette. Optical density was measured at 400 nm using a Novaspect-II spectrophotometer (France). The experiments were performed using three biological replicates.

### Permeability test

To assess the permeability of the *C. jejuni* envelope, strains were grown in BHI supplemented with 50 µmol/L hemin (Sigma-Aldrich) at 37°C overnight. Cultures were pelleted by centrifugation and washed with PBS. The OD_600_ was adjusted to 0.5 with PBS, and 200 µL aliquots were distributed to a 96-well cell culture plate. Ethidium bromide was added to the wells at a final concentration of 1 µg/mL (85). Fluorescence was measured using a Tecan Spark multimodal reader (Tecan Life Sciences, France) with 530 nm band-pass and 600 nm high-pass filters as the excitation and detection wavelengths, respectively. Fluorescence was measured every 60 s for 120 min at 37°C. All assays were performed in triplicate.

### bEV isolation and purification

bEVs were collected and purified from unstressed and stressed *C. jejuni* culture supernatants using a protocol adapted from reference 86. Briefly, bacterial cells were removed by centrifugation at 5,750 × *g* for 5 min and filtered through a 0.22 µm membrane filter. The supernatant was then ultra-centrifuged at 120,000 × *g* for 2 h in a Beckman XL-90 ultracentrifuge with an SW41 rotor to pellet the bEVs. The resulting vesicle pellet was suspended in PBS and then added to a three-layer iodixanol discontinuous gradient of 45%, 26%, and 10% (wt/vol) in SM Buffer (50 mM Tris pH 7.5, 100 mM NaCl, and 10 mM MgCl_2_). Tubes were then ultra-centrifuged at 200,000 × *g* for 5 h in a Beckman XL-90 ultracentrifuge with an SW55 rotor, and purified bEVs were collected and stored at 4°C.

The concentration of bEVs was determined by the rate of Brownian motion using a ZetaView particle tracking analyzer (Excilone Services, France) equipped with fast video capture and particle-tracking software (ZetaViewer, Excilone Services, France). The samples were diluted in PBS before analysis (usually 1:50), and 1 mL of the diluted bEV solution was discharged into the chamber of the system. The size distribution of bEV samples was characterized using dynamic light scattering (DLS) with a Zetasizer Pro (Malvern Panalytical, Malvern, UK). The scattering intensity data were processed using instrument software to obtain the size distribution of the particles. High-sensitivity flow cytometry for nanoparticle analysis (NanoFCM) was used to assess the purity, concentration, and mean size of EV samples. This was performed on a NanoFCM flow nanoanalyzer (NanoFCM Co.) following the manufacturer’s instructions to confirm the particle size. The instrument was calibrated for particle size with 200 nm polystyrene beads and a silica nanosphere mixture (pre-mixed silica beads with diameters of 68, 91, 113, and 151 nm, currently called “NanoFCM Quality Control Nanospheres,” provided by nanoFCM). Each bEV preparation (20 µL) was diluted with sterile PBS in the 1:10–1:2,000 range, allowing the detection of at least 500 particles per acquisition. Events were recorded twice for each biological sample for 60 s. The flow rate and side scattering intensities were converted into the corresponding particle sizes using nanoFCM software. MemGlow 488 (Cytoskeleton, USA) labeling was used to assess bEV purity. A Live/dead Bacterial Viability kit was used to observe the presence of internal (Syto9) and external (propidium iodide) DNA carried by the bEVs.

### Vesicle imaging and dry mass measurement

Dry mass measurements were carried out using a custom-made quantitative phase imaging system, under the assumption that optical phase distortions can be considered proportional to the dry mass, that is, the mass of the solute without water (39, 87). This quadriwave lateral shearing interferometry (QLSI) setup, based on an inverted microscope (Olympus IX-71), is described in reference 40 and in supplementary data for details. The samples were then imaged on a custom-made high-resolution wavefront sensor (WFS) based on QLSI (87). A two-dimensional 20 μm-step 0−π phase-only checkerboard grating QLSI mask (PhiMask, Idylle) was reimaged a few millimeters (*d* = 1.6 mm) before the camera. To reduce the system vibrations, we used a passively cooled (i.e., no fan) ZWO ASI174MM camera.

### Imaging of bacterial cells and bEVs by TEM

Samples of bacteria were fixed with 2% glutaraldehyde in 0.1 M Na cacodylate buffer pH 7.2 and contrasted with Oolong Tea Extract (OTE) 0.2% in cacodylate buffer, postfixed with 1% osmium tetroxide containing 1.5% potassium cyanoferrate, gradually dehydrated in ethanol (30%–100%) and substituted gradually in a mix of ethanol-epon and embedded in Epon (Delta microscopie, France). Thin sections (70 nm) were collected on 200 mesh copper grids and stained with lead citrate. The grids were examined with a Hitachi HT7700 electron microscope operated at 80 kV (Milexia, France), and images were acquired using a charge-coupled device camera (AMT).

For TEM imaging of bEVs, they were fixed with 2% glutaraldehyde in 0.1 M Na cacodylate buffer (pH 7.2), directly adsorbed onto a carbon film membrane on a 300-mesh copper grid, stained with 1% uranyl acetate dissolved in distilled water, and dried at room temperature. The grids were examined with a Hitachi HT7700 electron microscope operated at 80 kV (Milexia, France), and images were acquired using a charge-coupled device camera (AMT).

### Imaging of bacterial cells by SEM

After overnight incubation, 2 mL of culture was gently pelleted (2,000 × *g*, 4 min) to 50 µL, which was fixed in 2 mL 2% glutaraldehyde buffered with sodium cacodylate (0.2 M) for 2 h at room temperature, then overnight at 4°C, in a 24 multi-well plate (TPP 92024, Switzerland) that contained 1 cm × 1 cm glass slides at the bottom of the well. Samples attached to the glass slides were rinsed twice for 5 min in 0.2 M sodium cacodylate buffer, then in successive baths of ethanol (50%, 70%, 90%, 100%, and anhydrous 100%), and finally dried using a Leica EM300 critical point apparatus with slow 20 exchange cycles, with a 2-min delay. The samples were mounted on aluminum stubs with adhesive carbon (EMS, LFG France) and coated with 6 nm of Au/Pd using a Quorum SC7620, 50 Pa of Ar, and 180 s of sputtering at 3.5 mA. Samples were observed using the SE detector of a FEG SEM Hitachi SU5000, 2 keV, 30 spot size, 5 mm working distance, located on the MIMA2 core facility, INRAE, Jouy-en-Josas, France (https://doi.org/10.15454/1.5572348210007727E12).

### Proteomic studies

For proteome analysis, the samples were concentrated 6.5 times on 10 kDa filters to allow for protein quantification by the Bradford assay using a protein assay kit (Thermo Fisher Scientific) following the manufacturer’s protocol. Absorbance was measured using a spectrophotometer (Novaspec III, Biochrom) at 595 nm. Then, aliquots of 40 µg of protein were processed using a modified filter-aided sample preparation (FASP) protocol for proteomic analysis (88). Briefly, bEV samples were diluted and incubated for 15 min in 200 µL of lysis buffer (8M urea, 25 mM HEPES, pH 8.0, 5% glycerol, 1 mM DTT, 0.2% DDM, and 1:200 vol/vol protease inhibitor) before being transferred to a 10 kDa molecular weight cut-off (MWCO) filter (MRCPRT010, Millipore). The sample volume was reduced to 50 µL prior to the addition of 100 µL denaturation buffer (8M urea, 25 mM HEPES, pH 8.0). The sample volume was reduced to 20 µL by centrifugation at 14,000 × *g*. After that, proteins were reduced by adding 4 mM Tris(2-carboxyethyl)phosphine (TCEP) (Thermo Fisher Scientific) in denaturation buffer and incubating for 30 min at RT followed by centrifugation for 30 min at 14,000 × *g*. Proteins were then alkylated using a 40-min RT incubation with 20 mM iodoacetamide (IAA) in 100 µL denaturation buffer, followed by a 30-min centrifugation at 14,000 × *g*. Next, digestion buffer was added (0.6% vol/vol glycerol, 25 mM HEPES, pH = 8.0), and the remaining urea was washed by a centrifugation step before the addition of MS-grade trypsin/Lys-C mix (Promega) with a 1:150 enzyme-to-protein ratio. The proteolytic digestion was performed in the dark under agitation (600 rpm) at 37°C for 12 h. Peptides were eluted as filtrate by centrifugation at 14,000 × *g* for 30 min, and then acidified with 2% (vol/vol) formic acid and desalted for MS analysis using Pierce C-18 spin columns (Thermofisher Scientific). Purified peptides were vacuum-dried (Savant SPD111V SpeedVac Concentrator, Thermo Scientific) and reconstituted with 20 µL of MS-grade water with 0.1% formic acid for MS analysis.

Proteomic analysis was performed as previously described (89). Approximately 4  µg of protein, quantified using the Bradford assay, was injected into an UltiMate 3000 nanoRSLC (Dionex, Thermo Fisher Scientific, Mississauga, ON, Canada) coupled to an Orbitrap Fusion mass spectrometer (Thermo Fisher Scientific, Mississauga, ON, Canada) for proteomic analysis. The mass spectrometry data were processed for protein identification and quantification using MaxQuant 2.6.4.0 (90). The acquired peptide spectra were matched against the *C. jejuni* protein database from NCBI (taxonomy ID: 197), 1,737 reviewed and 78,037 unreviewed entries as of 10.10.2024.

Quantitative bioinformatics analysis of *C. jejuni* whole-cell samples was conducted using the R programming language in RStudio (2024.04. v2). To visualize common and unique proteins, Venn diagrams were created using the ggVenn package in R, where unique proteins were defined as those present in at least 2/4 samples of a particular group. Principal component analysis was conducted to comparatively visualize the entire proteome. Missing LFQ intensity data were first imputed. The data were then log2 normalized and scaled. The components were then calculated using the prcomp() function in the stats package. Principal component 1 (PC1) and principal component 2 (PC2) were plotted using the ggplot2 package.

Additionally, a comparison of the log_2_ normalized data to common proteins was performed by conducting a Student’s *t*-test with the *t*-test () function from the stats package. The log2 fold change was also calculated. Both *P*-value and fold change parameters for stressed and non-stressed common proteins from *C. jejuni* whole cells were plotted to create a volcano plot. Overall protein significance was defined as a *P* value <0.05, and a minimum fold change (stressed/non-stressed) of 1.5.

*C. jejuni* bEV samples were analyzed using RStudio software. The data were imported and filtered, as described above. A Venn diagram was also created for the bEV using the ggVenn package, as described above. KEGG pathway enrichment analysis was also conducted using the proteins identified under stressed and non-stressed conditions using the Eggnog mapper 2.1.12. First, IDs were mapped using the UniPort ID mapping tool (https://www.uniprot.org/id-mapping). The results were downloaded and compressed into a FASTA file and uploaded into EggNog Mapper (http://eggnog-mapper.embl.de/) for KEGG pathway analysis.

### Lipidomic study

To obtain lipid fractions, bacterial cells or purified bEVs were freeze-dried and extracted with 4.75 mL of chloroform-methanol 0.3% NaCl (1:2:0.8 vol/vol/vol) at 80°C for 15 min. The solutions were vortexed for 1 h at room temperature and centrifuged at 4,000 rpm for 15 min at room temperature. Supernatants were collected, and the debris was re-extracted with 9.5 mL of the same mixture by vortexing for 30 s. After the second centrifugation, supernatants were pooled and diluted with 2.5 mL each of chloroform and 0.3% NaCl solution. Phase separation was achieved by centrifugation at 4,000 rpm for 15 min at room temperature. The upper phase was discarded, and the chloroform phase was evaporated to dryness under a nitrogen stream and stored at −20°C.

Phospholipid analysis was performed using a Dionex Ultimate 3000 RSLC system (Thermo Fisher Scientific) equipped with two quaternary pumps, an autosampler, and a column oven. The RSLC system was coupled online to an LTQ Orbitrap Velos Pro mass spectrometer equipped with a linear ion trap and orbital trap analyzer (Thermo Fisher Scientific, Waltham, MA, USA). Lipid separation was performed as described previously (43). Prior to injection into the chromatographic system, dry pellets were solubilized in appropriate volumes of chloroform to achieve comparable concentrations (60 mg/mL for bacterial samples) and in a minimal volume of 50 µL for bEVs (6 mg/mL–10 mg/mL bEVs). Phospholipids were separated according to their polarity; the retention time provides information on the polar head group, which is compared to commercial standards. Individual species were identified for each chromatographic peak. Phospholipids were identified in negative electrospray ionization mode (ESI−) using high-resolution mass detection in full-scan MS1 mode on the Orbitrap. The exact mass obtained in MS1 was used to confirm the class of phospholipids and to determine the number of carbons and amount of unsaturation of each m/z. Data-dependent MS2 fragmentation was also conducted in a linear ion trap to determine the composition of the fatty acyl chains. MSn fragmentation is not always possible because of the low abundance of some ions. In these cases, phospholipids are referred to as PL(*X*:*n*), where PL is the phospholipid class, *X* represents the total number of carbon atoms in the fatty acyl chains, and *n* is the total number of unsaturations. Phospholipids have been reported to have a specific fatty acyl chain composition, for example, PL(16:0-18:1). The sn-1 and sn-2 positions of acyl chains were not determined.

### Fatty acid identification

FA profiles were analyzed using two methods. The first method was described previously (91). Briefly, bacteria were harvested by centrifugation (5,000 × *g*, 5 min), and purified bEVs were freeze-dried before storage at −80°C until further processing. Extraction and trans-esterification of FA were performed directly on bacterial pellets or lyophilized bEVs. Gas chromatography separation was performed by injection of the FA methyl esters in a split-splitless mode of an AutoSystemXL gas chromatograph (PerkinElmer) equipped with a ZB-Wax capillary column (30 m × 0.25 mm × 0.25 mm; Phenomenex) and a flame ionization detector. FAs were identified based on their retention times. Data were analyzed using the TotalChrom Workstation (PerkinElmer). The results are presented as representative gas chromatography profiles and as FA peak areas (expressed as a percentage of the total areas of detected peaks).

The second method was used because it allows the extraction of all FA, including hydroxylated FA (92, 93). Briefly, whole-cell FA were first saponified and esterified using methanolic NaOH (1 mL of 3.75 M NaOH in 50% [vol/vol] methanol and incubated for 30 min at 100°C), followed by treatment with methanolic HCl (addition of 2 mL of 3.25 M HCl in 45% [vol/vol] methanol solution and incubation for 10 min at 80°C). Fatty acid methyl esters (FAME) were extracted using a 1:1 (vol/vol) diethyl ether/cyclohexane mixture. The organic phase was washed with a dilute base solution (0.3 M NaOH). Analytical gas chromatography of FAME was carried out in a GC-MS Trace 1300/ISQ 7000 system (Thermo Fisher Scientific) equipped with a BPX70 capillary column (25 m, 0.22 mm internal diameter; SGE, Victoria, Australia). The column temperature was initially maintained at 100°C for 1 min, then gradually increased to 170°C at a rate of 2°C per min. FA species were identified based on mass spectrometry (MS) databases (Replib, Mainlib, FAME2011) (94). The relative abundance of each FA was calculated as the percentage of the total FAME peak area. The identified FA species were grouped into the following categories: saturated FA (SFA), unsaturated FA (UFA), and cyclopropane FA (cycloFAs).

### Membrane fluidity test

The generalized polarization of the lipophilic dye Laurdan (6-dodecanoyl-2-dimethylaminonaphthalene) when bound to *C. jejuni* plasma membrane was used as a measure of bacterial fluidity (95). Bacteria were sampled (1 mL), collected by centrifugation (5,000 × *g*, 5 min), resuspended in PBS, and incubated for 10 min in the dark with 10 µM Laurdan (Sigma-Aldrich) from a 1 mM stock in dimethylformamide (DMF). Then, unbound Laurdan was removed using four cycles of centrifugation (8,000 × *g*, 5 min) and resuspended in pre-warmed (30°C) 1% (vol/vol) DMF in mineral water (by vortexing). After final resuspension, technical replicates (200 µL) were added to a pre-warmed (30°C) clear-bottom black 96-well plate, and Laurdan fluorescence was induced at 350 nm and recorded at 450 and 500 nm using a Tecan Spark (Tecan Life Sciences) microplate reader. The general polarization (GP) of Laurdan was determined using the formula: GP = (*I*_450_ − *I*_500_)/(*I*_450_ + *I*_500_), where *I* corresponds to the fluorescence intensity value at the recorded emission wavelength (95). An increase of Laurdan GP values corresponds to a reduction in membrane fluidity (rigidification).

### Caco-2 cell culture and cytotoxicity evaluation

The human colorectal carcinoma cell line (Caco-2, ATCC HTB-37) was cultured following ATCC recommendations for this cell line, with minor modifications. Caco-2 cells were routinely cultured in DMEM GlutaMAX growth medium (Gibco, Thermo Fisher Scientific, USA) supplemented with 20% fetal calf serum (FCS, Gibco, France), 1% penicillin-streptomycin (Sigma-Aldrich, USA) in T25 cell culture flasks at 37°C in a 5% CO_2_ humidified atmosphere. The cells were passaged every 2–3 days. For use in the assays, Caco-2 cells were seeded in 96-well culture plates at a density of 3 × 10^4^/well and grown to confluence before further treatment.

The cytotoxic effect of bEVs and culture supernatants from *C. jejuni* Bf on the viability of cultured Caco-2 cells was determined using the MTT assay. Caco-2 cells were seeded in 96-well plates (Stardedt) and grown for 48 h to confluence before the addition of *C. jejuni* supernatant and bEVs (MOI 20). The blank contained only the medium, whereas the positive control contained cells and medium. Supernatants were added at a final concentration of 20% in complete culture medium, and the plate was incubated for 24 h at 37°C in a 5% CO_2_ atmosphere. After incubation, the medium was replaced with 100 µL of medium containing MTT reagent (1 mg/mL) and incubated at 37°C in a 5% CO_2_ atmosphere for 1 h. Subsequently, the medium was removed, the plate was dried for 15 min, and 100 µL of DMSO was added to dissolve the formazan crystals formed. The absorbance of the plate was measured at 670 nm (for background measurements) and 560 nm using a microplate reader (Tecan Spark). Cell viability was calculated by removing the blank value from all samples and normalizing treated cells to untreated cells (representing 100% cell viability).

The TEER values reflect the integrity of a given monolayer and are indicative of the polarization of epithelial cells. The TEER across the monolayer of Caco-2 cells was measured first to determine confluency and then to evaluate the cytotoxic effects of *C. jejuni* bacteria and bEVs on intestinal epithelial cells. For the TEER experiment, Caco-2 cells were seeded in 24-well Transwell plates (Corning Costar, 8 µm pore size, PET membrane, SURF) and grown for 5 days to confluence before the addition of *C. jejuni* cells and bEVs. TEER values between 150 and 600 Ω × cm^2^ were considered indicative of a confluent monolayer, and the experimental values observed ranged between these values.

### Statistics

Statistical analyses were performed using GraphPad Prism 10.2.1 (GraphPad Software). Data obtained from experiments performed with three independent biological replicates (*n* = 3) are presented as mean ± standard deviation or median ± interquartile range. *P* ≤0.05 were considered significant for all analyses performed and are indicated with asterisks: *P ≤0.05, **P ≤0.01, and ***P ≤0.001. Additional test details can be found in the Source data file.

## Data Availability

The mass spectrometry proteomics data were deposited in the ProteomeXchange Consortium via the PRIDE partner repository with the data set identifier PXD074845.

## SUPPLEMENTAL MATERIAL

Supplemental figures aem.01265-26-s0001.docx Fig. S1 to S5.

Supplemental legends aem.01265-26-s0002.docx Legends for Videos S1 and S2.

Source data aem.01265-26-s0003.xlsx Raw data associated with figures.

Table S1 aem.01265-26-s0004.xls Lipidomic data of cells and EVs.

Table S2 aem.01265-26-s0005.xlsx Proteomic data of cells and EVs.

Table S3 aem.01265-26-s0006.xlsx Raw TEER data and analysis.

Video S1 aem.01265-26-s0007.avi *Campylobacter jejuni* Bf cells were observed after cultivation under microaerophilic conditions.

Video S2 aem.01265-26-s0008.avi *Campylobacter jejuni* Bf cells were observed 4 h after exposure to combined thermal and oxidative stress.

ASM does not own the copyrights to Supplemental Material that may be linked to, or accessed through, an article. The authors have granted ASM a non-exclusive, world-wide license to publish the Supplemental Material files. Please contact the corresponding author directly for reuse.
