# Supplementary material for: Poultry handling-related stress alters membrane composition, extracellular vesicle production, survival, and virulence in Campylobacter jejuni Bf
Source: Appl Environ Microbiol. 2026 Aug 14;92(9):e01265-26. doi: 10.1128/aem.01265-26 (PMC13599532; doi:10.1128/aem.01265-26)
Supplement: Supplementary file 1 [file aem.01265-26-s0001.docx]

**Supplementary Information**

**Supplementary Figures**

**Poultry Handling-Related Stress Alters Membrane Composition, Extracellular Vesicle Production, Survival, and Virulence in *Campylobacter jejuni Bf***

Jeanne Malet-Villemagne^1^, Rochelle D’Mello^2^, Yingxi Li^2,^ Zoran Minic^2^, Karine Gloux^1^, Florence Dubois-Brissonnet^1^, Bastien Prost^3^, Audrey Solgadi^3^, Christine Péchoux^5^, Vlad Costache^1,5^, Marianne De Paepe^1^, Zhu Zhang^6^, Gilles Tessier^6,7^, Jasmina Vidic^1,*^

^1^Université Paris-Saclay, INRAE, AgroParisTech, Micalis Institute, Jouy en Josas, France.

^2^University of Ottawa, Department of Chemistry and Biomolecular Sciences, John L. Holmes Mass Spectrometry Facility, Ottawa, ON, Canada.

^3^UMS-IPSIT SAMM Facility, Université Paris-Saclay, Inserm, CNRS, Ingénierie et Plateformes au Service de l'Innovation Thérapeutique, Paris-Saclay, France.

^4^Université Paris-Saclay, INRAE, GABI, Jouy en Josas, France.

^5^MIMA2 Imaging Core Facility, Micalis Institute, INRAE, 78350 Jouy-en-Josas, France.

^6^Sorbonne Université, CNRS UMR7210, INSERM UMRS968, Institut de la Vision, 75012 Paris, France.

^7^Université Paris Cité, paris 75006, France.

* Corresponding author: [jasmina.vidic@inrae.fr](mailto:jasmina.vidic@inrae.fr)

Summary:

Figure S1: Isolation and purification protocol of EVs.

Figure S2: Fatty acid profiles of *C. jejuni* Bf cells identified by GC-MS.

Figure S3: Heat map of *C. jejuni* Bf proteins.

Figure S4: Optical microscopy images of Caco-2 monolayers on Transwell membranes before TEER measurement.

Figure S5: TEER (transepithelial electrical resistance) measurements of *C. jejuni* cells added on Caco-2 cells by the apical channel

**Fig. S1.** Isolation and purification protocol of EVs. After supernatant centrifugation, the pellet contains bEVs but also protein aggregates (A). To eliminate protein aggregates, the pellet is resuspended in PBS and added on a 3-layer (10%, 26%, 45%) discontinuous iodixanol gradient, shown in (B). Vesicles were found in fractions 2, 3, 4 and 5 (very few), as shown on the electronic microscopy pictures. Scale bars = 500 nm. Measurement of hydrodynamic diameter R_H_ of particles present in the supernatant before and after gradient by DLS confirm the elimination of protein contaminants from the vesicle sample (C). Finally, a MemGlow membrane staining of the particles analyzed in NanoFCM show a minimal purity of 85% (D).

**Fig. S2.** Fatty acid profiles of *C. jejuni* Bf cells after extraction using diethyl/cyclohexane (allowing extraction of hydroxylated FAs) and identified by GC-MS. Fatty acids profile of non-stressed (A) and stressed (B) bacteria are presented. The relative abundance of each fatty acid was calculated using peak area (in % of total peak area). Mean relative abundance (n=3) of fatty acids (C) and fatty acid groups (D) in non-stressed and stressed cells.

**Fig. S3.** Interactive cluster heatmap representing *C. jejuni* Bf proteins identified in non-stressed and stressed cells. 4 biological replicates were analyzed for each condition (n=4). Heat map was realized based on mean z-scored protein LFQ intensities of 1084 differentially expressed proteins (ANOVA FDR < 0.05) after unsupervised hierarchical clustering.

**Fig. S4.** Morphological evolution of Caco-2 monolayers on Transwell membranes before TEER measurement. Microscopic validation of cell confluence, obtained between day 4 and day 5. Brightfield images are presented with scale bars.

**(Ωxcm^2^)**

**Fig. S5.** TEER (transepithelial electrical resistance) measurements of *C. jejuni* non-stressed (NS) or stressed (S) cells added on Caco-2 cells by the basal channel. Error bars represent the SD from three independent experiments (n = 3). Confluence was reached at day 6, and is marked with a green arrow.
