# Supplementary material for: Poultry handling-related stress alters membrane composition, extracellular vesicle production, survival, and virulence in Campylobacter jejuni Bf
Source: Appl Environ Microbiol. 2026 Aug 14;92(9):e01265-26. doi: 10.1128/aem.01265-26 (PMC13599532; doi:10.1128/aem.01265-26)
Supplement: Supplementary file 2 [file aem.01265-26-s0002.docx]

**Supplementary Information**

**Supplementary Videos**

**Versatility of Campylobacter jejuni Bf extracellular vesicles in regulating adaptation and virulence under combined thermal and oxidative stress**

Jeanne Malet-Villemagne^1^, Rochelle D’Mello^2^, Yingxi Li^2,^ Zoran Minic^2^, Karine Gloux^1^, Florence Dubois-Brissonnet^1^, Bastien Prost^3^, Audrey Solgadi^3^, Christine Péchoux^5^, Vlad Costache^1,5^, Marianne De Paepe^1^, Zhu Zhang^6^, Gilles Tessier^6,7^, Jasmina Vidic^1,*^

^1^Université Paris-Saclay, INRAE, AgroParisTech, Micalis Institute, Jouy en Josas, France.

^2^University of Ottawa, Department of Chemistry and Biomolecular Sciences, John L. Holmes Mass Spectrometry Facility, Ottawa, ON, Canada.

^3^UMS-IPSIT SAMM Facility, Université Paris-Saclay, Inserm, CNRS, Ingénierie et Plateformes au Service de l'Innovation Thérapeutique, Paris-Saclay, France.

^4^Université Paris-Saclay, INRAE, GABI, Jouy en Josas, France.

^5^MIMA2 Imaging Core Facility, Micalis Institute, INRAE, 78350 Jouy-en-Josas, France.

^6^Sorbonne Université, CNRS UMR7210, INSERM UMRS968, Institut de la Vision, 75012 Paris, France.

^7^Université Paris Cité, paris 75006, France.

* Corresponding author: [jasmina.vidic@inrae.fr](mailto:jasmina.vidic@inrae.fr)

Summary:

Video S1:

*Campylobacter jejuni* Bf cells were observed after cultivation under microaerophilic conditions. The time-lapse optical microscopy video shows bacterial cells in BHI medium containing hemin. *C. jejuni* moves in solution using flagella, which rotate like tiny propellers driven by a proton motive force across the bacterial membrane. Due to its curved, spiral shape, the bacterium exhibits a distinctive corkscrew-like motion that allows it to move efficiently through liquides. This type of motility enables the organism to navigate its surroundings and plays an important role in its ability to reach, colonize, and infect host tissues. The selected video is a merge of images acquired with an Axio-Observer Z1 inverted microscope (Zeiss) equipped with an AxioCam 807 mono digital camera (Zeiss) and a ×100 Apochromat objective (Zeiss). The video was processed using the ZEN software package (Zeiss).

Video S2:

*Campylobacter jejuni* Bf cells were observed 4 hours after exposure to combined thermal and oxidative stress in BHI medium containing hemin. The time-lapse optical microscopy video shows that the normally smooth, corkscrew-like swimming (Video S1) becomes slower, less coordinated, and sometimes erratic. Some cells briefly exhibit increased tumbling or irregular motion, reflecting a stress response rather than purposeful movement. The selected video is a composite of images acquired with an Axio-Observer Z1 inverted microscope (Zeiss) equipped with an AxioCam 807 mono digital camera (Zeiss) and a ×100 Apochromat objective (Zeiss). The video was processed using the ZEN software package (Zeiss).
